# Pharmaceutical Market Analysis of Topical Semi-Solid Dosage Forms for Skin Disorders in Kazakhstan

**DOI:** 10.3390/ph19091357

**Published:** 2026-08-27

**Authors:** Artyom Savelyev, Dmitriy Khrustalev, Irina Losseva, Azamat Yedrissov, Anastassiya Khrustaleva, Vladimir Kazantsev, Sofiya Shapovalenko, Marlen Kiikbayev, Polina Rusyaeva, Kristina Perepelitsyna

**Affiliations:** School of Pharmacy, NPJSC “Karaganda Medical University”, 40 Gogol St., Karaganda 100008, Kazakhstan; savelev@qmu.kz (A.S.); loseva@qmu.kz (I.L.); anasteishin_2009@mail.ru (A.K.); shapovalenko@qmu.kz (S.S.); rusyaevapolina@mail.ru (P.R.);

**Keywords:** semi-solid dosage forms, topical drug delivery, dermatitis, wound healing, pharmaceutical market analysis, Kazakhstan, dihydropyrimidine-2(1H)-thione, topical formulations, pharmaceutical development

## Abstract

**Background:** Topical semi-solid dosage forms are crucial for treating inflammatory skin disorders, wounds, burns, and superficial infections. Despite their widespread clinical use, comprehensive pharmaceutical market analyses of registered topical medicines remain limited in Central Asia. The aim of this study was to characterize the pharmaceutical market of topical semi-solid dosage forms registered in the Republic of Kazakhstan, including their dosage-form distribution, pharmacotherapeutic composition, manufacturer structure, and structural diversity of active pharmaceutical ingredients (APIs), with particular attention to heterocyclic compounds. **Methods:** A cross-sectional descriptive analysis was conducted using the State Register of Medicinal Products of the Republic of Kazakhstan. Registration records of topical semi-solid dosage forms were identified according to predefined inclusion criteria and classified by dosage form, pharmacotherapeutic category, manufacturer, country of origin, and API chemical structure. Descriptive statistical analysis, market concentration indices, and structural classification of heterocyclic compounds were applied. Therapeutic recommendations for dermatitis management from selected CIS countries were reviewed to provide a contextual interpretation of the observed market structure. **Results:** A total of 1046 registered topical semi-solid dosage forms met the inclusion criteria. Ointments (39.5%), gels (28.9%), and creams (25.4%) accounted for 93.8% of all registered products, indicating a highly concentrated dosage-form structure. Anti-inflammatory agents represented the largest pharmacotherapeutic group, whereas wound-healing and combination products accounted for smaller proportions of the market. Structural classification demonstrated broad representation of established heterocyclic chemical classes, while no registered medicinal products containing dihydropyrimidine-2(1H)-thione (DHPM-thione) derivatives were identified in the registry. Comparison with regional clinical recommendations indicated differences between recommended therapeutic approaches and the distribution of registered pharmacotherapeutic categories. **Conclusions:** This study provides the first comprehensive descriptive assessment of the registered market of topical semi-solid dosage forms in Kazakhstan. The results establish a reference dataset describing the current pharmaceutical landscape, including dosage forms, therapeutic categories, manufacturers, and API chemical classes. The absence of registered DHPM-thione-containing medicinal products indicates that this chemical class is currently not represented in the national medicines registry. Together with previously published preclinical evidence, this observation supports consideration of DHPM-thione derivatives as a subject for future pharmaceutical research, although the present study does not permit conclusions regarding their clinical effectiveness, market demand, or future commercialization.

## 1. Introduction

Wound healing remains one of the most significant challenges in modern healthcare due to the high prevalence of chronic wounds, diabetic ulcers, burns, postoperative complications, and inflammatory skin diseases. According to various studies, chronic wounds affect up to 1–2% of the population in developed countries [1,2,3].

In Europe, wound care accounts for approximately 2–4% of total healthcare expenditures [4], whereas in the United States, chronic wounds affect approximately 8.5 million people and are associated with annual costs of 28–90 billion U.S. dollars [2,5].

Diabetic foot ulcers (DFUs) affect 19–34% of diabetic patients, with 50–60% complicated by infection and up to 20% resulting in amputation [6,7].

Complete healing within 12 weeks occurs in only 30–40% of cases, and recurrence reaches 65% at five years [7]. Five-year mortality exceeds 60% [8].

The problem of wound injuries is not limited to chronic ulcers. According to data from the World Health Organization, approximately 11 million burn injuries occur worldwide each year, resulting in about 180,000 deaths [9]. According to the Global Burden of Disease Study 2019, the number of new burn cases was 8.38 million, the number of deaths exceeded 111,000, and the total disease burden reached 7.46 million DALYs [10]. Surgical site infections also contribute substantially to the burden of complicated wounds, with a pooled 30-day incidence of approximately 11% reported in a large meta-analysis [11]. Furthermore, inflammatory skin diseases remain one of the most common human pathologies: according to various estimates, chronic non-viral inflammatory dermatoses affect 20–25% of the population, and at least one dermatological condition within the past 12 months has been reported in 43.35% of the adult population in Europe [12,13].

Delayed wound healing is a complex, multifactorial process involving persistent inflammation, microbial contamination, biofilm formation, oxidative stress, impaired angiogenesis, and defects in tissue regeneration [14,15]. Chronic wounds are characterized by a prolonged inflammatory phase, overproduction of reactive oxygen species (ROS), recurrent infections, impaired re-epithelialization, and reduced tissue vascularization [5,15]. It has been established that excessive ROS production not only accompanies the pathological process but also directly sustains microbial virulence and the formation of bacterial biofilms [16]. Similar mechanisms are observed in diabetic wounds, where hyperglycemia exacerbates the inflammatory response, oxidative stress, and susceptibility to recurrent infections [17].

In this regard, semi-solid dosage forms for topical application play a special role in modern therapy. Ointments, creams, gels, hydrogels, liniments, pastes, patches, and transdermal therapeutic systems deliver active pharmaceutical ingredients directly to the site of the lesion, reduce systemic exposure to medicinal substances, and enable a prolonged therapeutic effect [18]. Advances in pharmaceutical technology have shifted from traditional ointments to advanced platforms such as hydrogels, nanostructured carriers, and 3D-printed dressings [17,18,19], while environmental factors, including air pollution and ultraviolet radiation, may further impair skin barrier function and delay tissue repair, thereby increasing the need for effective topical therapeutic strategies [20,21]. Current trends in wound care indicate a shift from single-component preparations to multifunctional systems that combine antibacterial, anti-inflammatory, antioxidant, and regenerative properties [14,22,23]. For example, experimental studies have shown that the CPO/Mg^2+^ composite hydrogel provides a bacteriostatic effect of more than 95% against Staphylococcus aureus and Pseudomonas aeruginosa and promotes the closure of more than 95% of infected wounds within 14 days [24]. Similarly, 3D-printed dressings containing a combination of curcumin and dipotassium glycyrrhizinate reduced *S. aureus* adhesion by 89.9% after 4 h and by 98.9% after 24 h [25]. However, it should be noted that most such studies have been conducted at the preclinical level, and clinical evidence of the superiority of multifunctional systems over traditional therapy remains limited [1,26].

Heterocyclic compounds, particularly pyrimidine and dihydropyrimidine derivatives, are of interest due to their broad pharmacological activities, including anti-inflammatory and antimicrobial effects [27,28,29].

Among heterocyclic compounds, 3,4-dihydropyrimidine and 3,4-dihydropyrimidine-2(1H)-thione derivatives have been investigated extensively in recent years. This class has demonstrated a broad spectrum of pharmacological activity, including anti-inflammatory, antioxidant, antimicrobial, and cytoprotective effects in preclinical studies [30,31,32]. Several DHPM derivatives have been reported to inhibit key inflammatory mediators, including mPGES-1, 5-LOX, COX-2, TNF-α, IL-6, and other signaling pathways involved in chronic inflammation and impaired tissue repair processes.

In addition, a number of dihydropyrimidine-2(1H)-thione derivatives have demonstrated pronounced antioxidant properties, which is of particular interest for the treatment of chronic wounds characterized by excessive formation of reactive oxygen species [33,34].

The combination of anti-inflammatory, antioxidant, and potential antimicrobial effects makes dihydropyrimidine derivatives potential subjects for further pharmaceutical research, particularly for the development of semi-solid dosage forms intended for the treatment of inflammatory and infected skin lesions. An additional advantage of this chemical class is the ability to obtain target compounds using efficient and scalable synthetic methods, including modified versions of the Biginelli reaction and microwave-assisted synthesis, which are consistent with modern principles of resource-efficient and “green” chemistry [35,36,37,38].

In recent years, the Republic of Kazakhstan has implemented several initiatives aimed at improving the country’s drug safety, developing domestic pharmaceutical production, and reducing dependence on imported medicines. According to data from the State Register of Medicines of the Republic of Kazakhstan, as of June 2026, 6530 medicinal products were registered, including 5618 products registered under the national procedure and 912 products registered in accordance with the rules of the Eurasian Economic Union. At the same time, the structure of the market for semi-solid dosage forms, the availability of wound-healing, antimicrobial, and anti-inflammatory drugs, as well as the representation of heterocyclic compounds, have not yet been subject to a comprehensive analysis. To the best of our knowledge, the available scientific literature lacks studies devoted to a comprehensive assessment of the market for semi-solid dosage forms in the Republic of Kazakhstan, including an analysis of the pharmacotherapeutic structure, the chemical nature of active pharmaceutical ingredients, and the registration status of various chemical classes.

The aim of this study was to conduct a descriptive analysis of semi-solid dosage forms registered in the Republic of Kazakhstan, with particular attention to the pharmacotherapeutic structure, the representation of heterocyclic compounds, and the registration status of dihydropyrimidine and dihydropyrimidine-2(1H)-thione derivatives.

To the best of our knowledge, this is the first comprehensive registry-based assessment of topical semi-solid dosage forms registered in Kazakhstan that integrates multiple analytical dimensions, including dosage-form distribution, pharmacotherapeutic composition, manufacturer geography, quantitative market structure, and structural–chemical classification of active pharmaceutical ingredients.

This integrated analytical framework extends beyond a conventional market survey by providing a systematic characterization of the registered pharmaceutical portfolio from technological, therapeutic, and chemical perspectives. The analysis identified differences in the representation of major pharmacotherapeutic categories, demonstrated the distribution of heterocyclic chemical classes within registered products, and confirmed that dihydropyrimidine-2(1H)-thione derivatives are currently not represented in the national medicines registry. Although pharmacological activities of this chemical class have been described in published preclinical studies, the present work does not evaluate their pharmaceutical performance or clinical potential.

Furthermore, comparison of the registered market structure with clinical recommendations from selected CIS countries provided regional context for interpreting the distribution of therapeutic categories.

Although the study is descriptive in nature and does not assess clinical effectiveness, healthcare utilization, or market demand, it establishes a reproducible reference dataset and an analytical framework that may support future pharmaceutical market research, formulation development, and regulatory decision-making in Kazakhstan and other Central Asian countries with comparable pharmaceutical systems.

Unlike conventional registry inventories, the present study integrates technological, pharmacotherapeutic, manufacturing, and structural-chemical analyses within a single analytical framework. This multidimensional approach enables characterization of the registered pharmaceutical portfolio from complementary perspectives and provides a reproducible methodology applicable to comparative studies of pharmaceutical markets in other countries.

## 2. Results

### 2.1. General Overview of the Market for Semi-Solid Dosage Forms in the Republic of Kazakhstan

**Figure 1:**
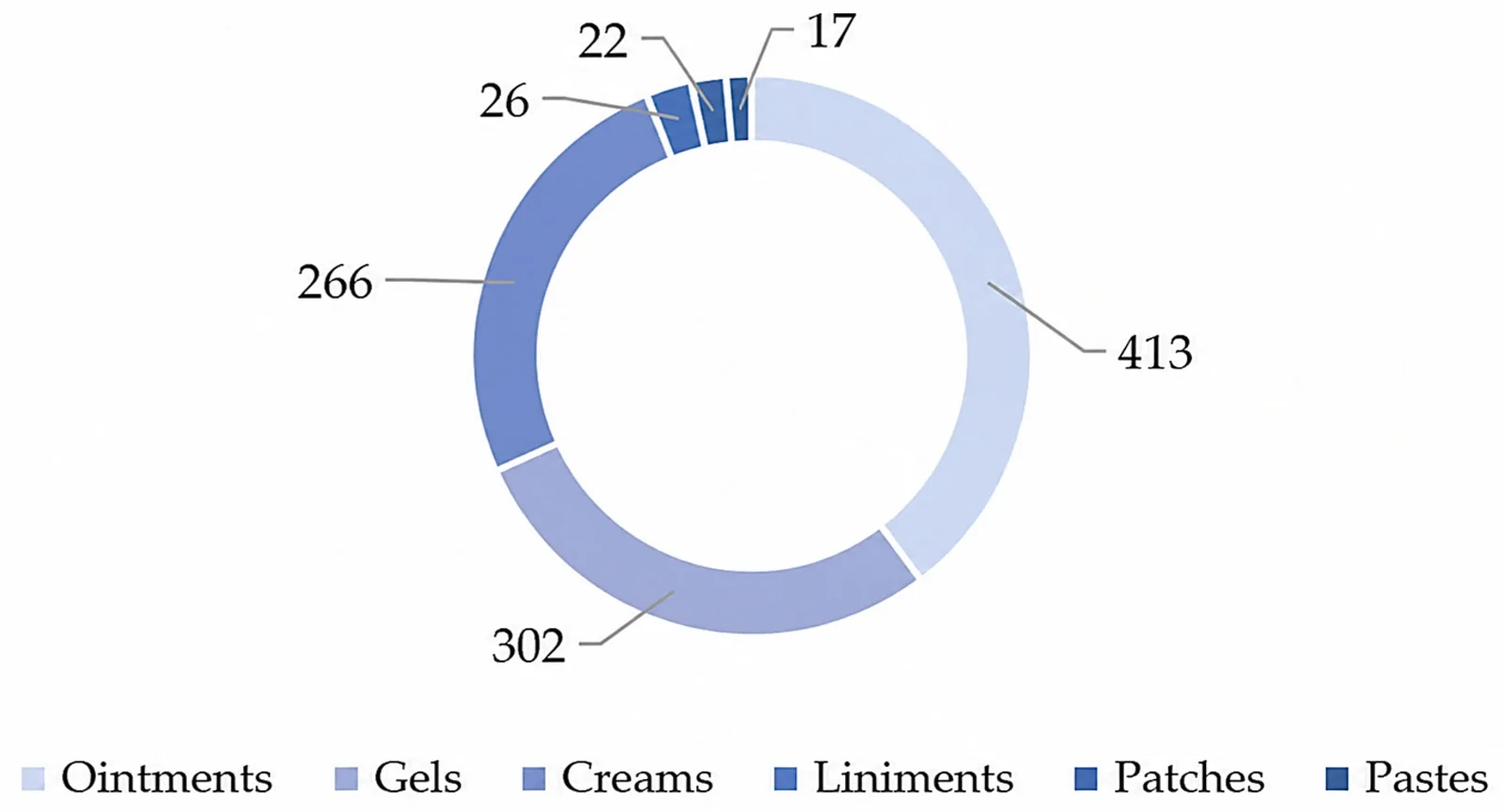
Pie chart of the semi-solid dosage forms market structure in the Republic of Kazakhstan.

As of June 2026, the State Register of Medicines of the Republic of Kazakhstan contained 6530 valid registration certificates, including 5618 drugs registered under the national procedure and 912 drugs registered under EAEU regulations. The distribution of the included semi-solid dosage forms by dosage form is presented in Figure 1.

Of 6530 registered products, 1046 topical semi-solid forms met the inclusion criteria. Table 1 shows the distribution: ointments (39.5%), gels (28.9%), creams (25.4%), with MCR_3_ = 93.8%.

The market for topical dosage forms in the Republic of Kazakhstan is characterized by a pronounced concentration around three main dosage forms—ointments, gels, and creams (Table 1)—indicating a high degree of standardization in topical therapy and limited representation of alternative drug delivery systems, including patches, transdermal therapeutic systems, specialized polymer carriers, and modern programmable plates and 3D-printed films [39]. At the same time, an analysis of the State Register of Medicines has shown that the market is characterized by a high degree of international diversification among manufacturers, with registered products originating from Kazakhstan, Russia, India, European countries, and several other regions.

Unlike segments focused on local production, the market for semi-solid dosage forms in the Republic of Kazakhstan remains predominantly import-dependent. Drugs of Russian origin are the most common, Russian manufacturers constituted the largest group of registered products in the dataset, similar regulatory requirements, and a wide range of generic topical drugs. Manufacturers from the European Union and India also account for a significant share of the market. The geographical structure of manufacturers is summarised in Table 2.

This approach preserves the proportional structure reported in the literature while providing a single consistent set of values, and the resulting structure is consistent with the overall import dependency of the Kazakhstani pharmaceutical market (86.9% imported products) [40] and with regional patterns observed in comparable topical product markets [42,43,44]. Among domestic manufacturers, the registry includes companies such as Khimfarm (Santo, a member of the Polpharma Group), Farmatsiya 2010, and several others; however, their registered product portfolios were predominantly composed of traditional ointments and a limited number of other topical dosage forms. Despite the diversity of manufacturers and countries of origin, further analysis showed that the registered products were based on a relatively limited range of chemical classes.

An analysis of the registry showed that approximately 83% of registered semi-solid dosage forms are supplied by foreign manufacturers. The market structure by origin of active pharmaceutical ingredients is presented in Table 3.

Russian companies constitute the largest market segment, while the share of domestic manufacturers remains substantially lower than the share of imported products. The estimated structure of the domestic segment of the semi-solid dosage forms market is presented in Table 4.

### 2.2. Assessment of Market Concentration and Technological Maturity

The market concentration ratio for the three leading dosage forms (MCR_3_) was:

MCR_3_ = 93.8%

which corresponds to a very high level of market concentration.

The share of modern dosage forms (gels, creams, patches) was 56.4%, while traditional forms (ointments, liniments, and pastes) accounted for 43.6%.

The calculated TMI was 56.4%, indicating that 56.4% of registered dosage forms belonged to the category operationally classified as modern within the descriptive framework of the present study. This result should be interpreted as a structural characteristic of the registry rather than evidence of technological innovation or pharmaceutical advancement.

The Formulation Innovation Ratio (FIR) was:

FIR = 1.29

which indicates that modern dosage forms are slightly more prevalent than traditional ones.

The key indicators of market structure and technological maturity are summarised in Table 5.

The Shannon index value (*H* = 1.26) indicates moderate structural diversity in the market. Despite the presence of six dosage-form categories, the distribution was highly uneven, with ointments, gels, and creams accounting for 93.8% of all registered products.

### 2.3. Pharmacotherapeutic Market Structure

An analysis of the pharmacotherapeutic profile revealed a marked predominance of anti-inflammatory drugs over wound-healing agents, as shown in Table 6.

**Figure 2:**
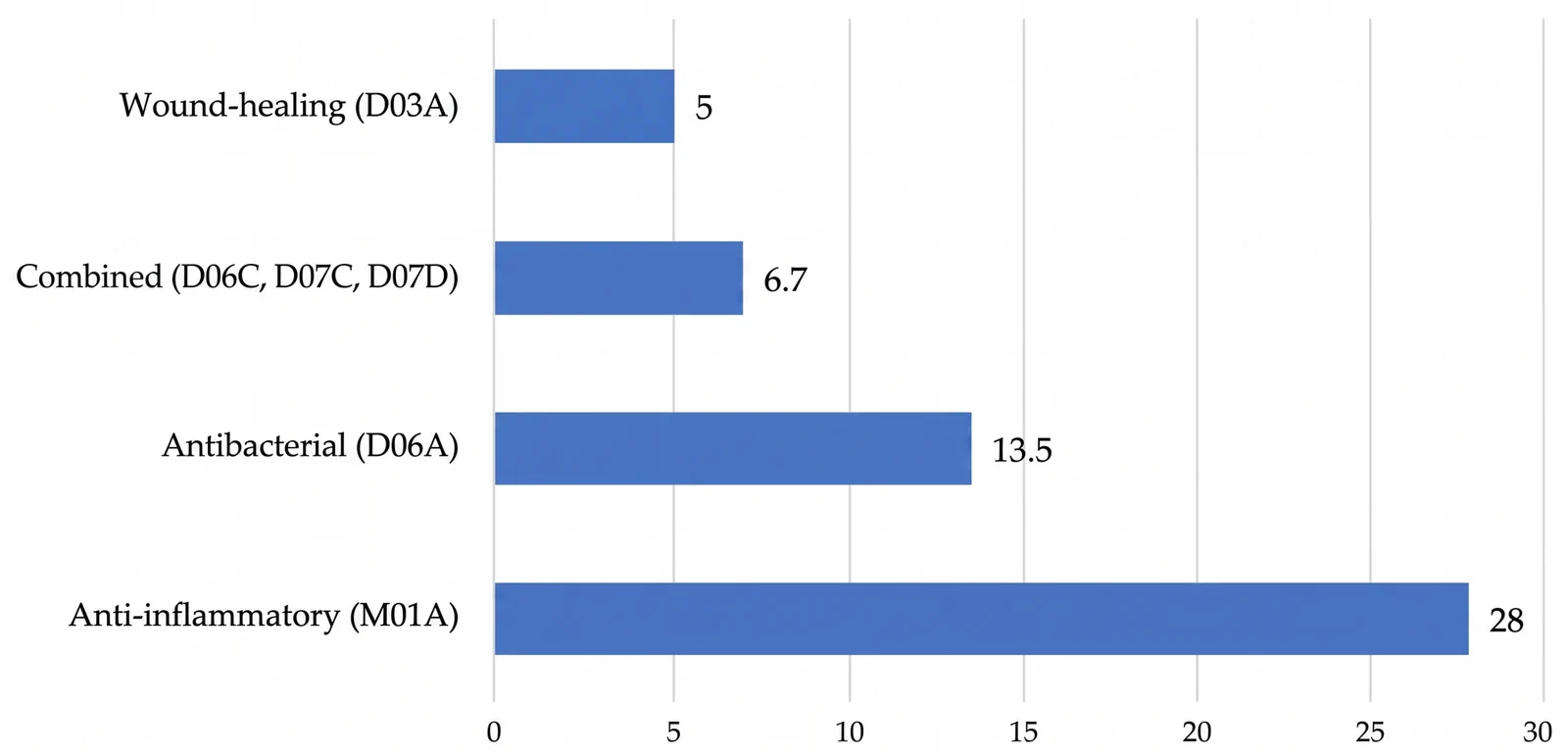
Distribution of main therapeutic categories among semi-solid dosage forms registered in the Republic of Kazakhstan.

The results demonstrate a marked predominance of anti-inflammatory products, with a comparatively lower representation of drugs aimed at stimulating tissue regeneration. This difference in representation was further quantified using the Therapeutic Gap Ratio (TGR), as presented in Table 7. Figure 2 illustrates the distribution of the principal pharmacotherapeutic categories.

### 2.4. Assessment of Differences in Therapeutic Representation

The difference in representation between anti-inflammatory and wound-healing drugs was:

TGR(AntiInfl → WoundHeal) = 5.6

This means that anti-inflammatory drugs were represented 5.6 times more frequently than wound-healing agents.

For antibacterial drugs relative to wound-healing agents, the corresponding figure was:

The results indicate that wound-healing products were less frequently represented than anti-inflammatory and antibacterial products in the registered portfolio.

The data indicate a difference in representation between therapeutic categories. The lowest proportional representation was observed for wound-healing products, while anti-inflammatory products were the most frequently represented category.

### 2.5. Structural and Chemical Profile of the Market

Among the 1046 registered topical semi-solid dosage forms, a substantial proportion contained heterocyclic active pharma-ceutical ingredients (APIs). The distribution of registered semi-solid dosage forms by chemical class is presented in Table 8.

The structural-chemical analysis showed that heterocyclic compounds represent a substantial proportion of registered APIs. Among the identified heterocyclic classes, imidazole derivatives (12.6%), triazole derivatives (6.5%), and pyrimidine derivatives (2.0%) were the most frequently represented groups. This indicates that DHPM-thione derivatives are not represented in the national medicines registry.

### 2.6. Comparison with Clinical Approaches to Dermatitis Therapy in CIS Countries

**Figure 3:**
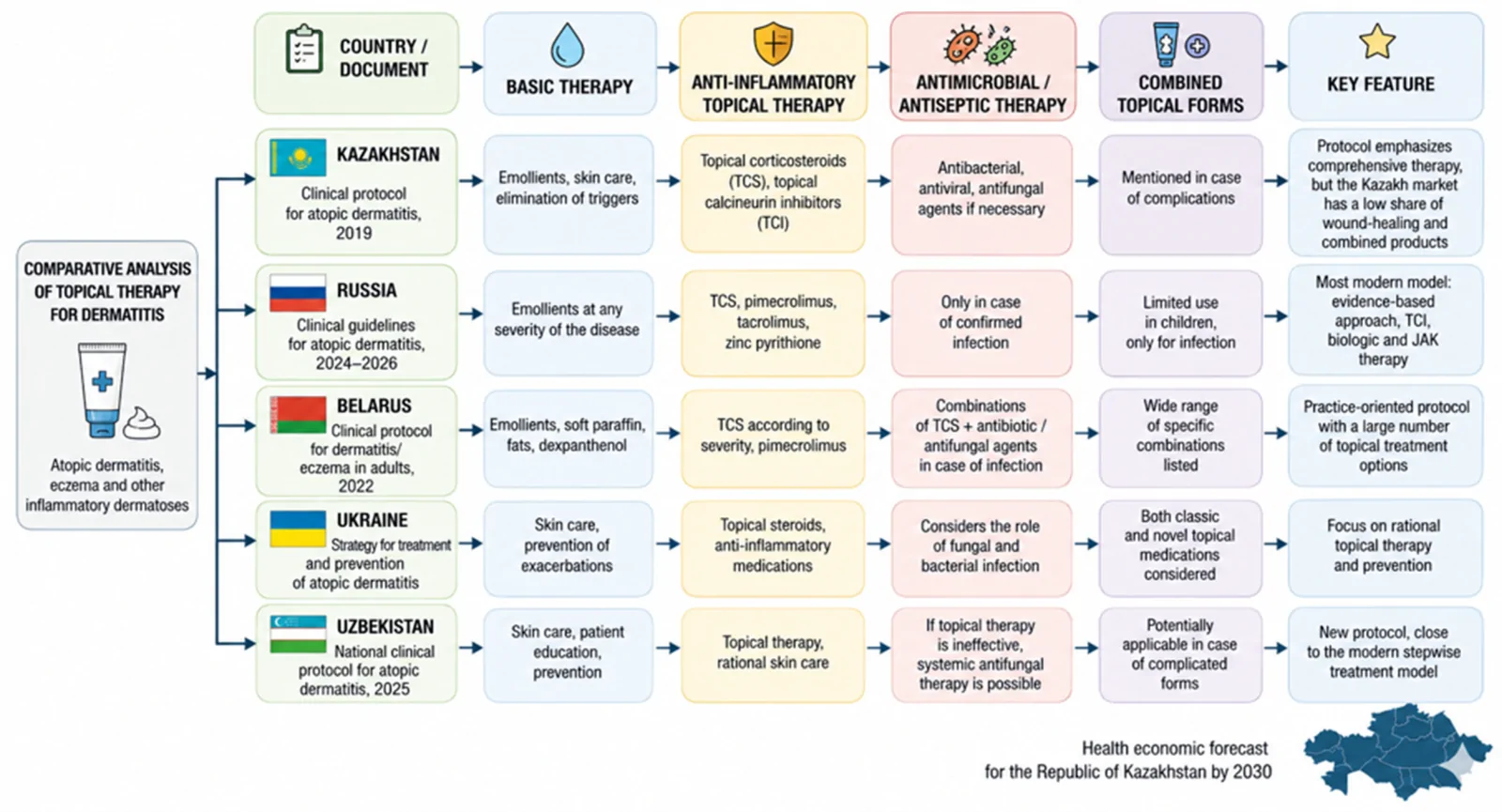
Comparative analysis of clinical approaches to external therapy of dermatitis in the CIS countries. The diagram was prepared using ChatGPT Plus 5.5 (OpenAI) solely for visualization of the summarized data.

To provide context for the observed market structure, a comparative analysis was conducted of approaches to the topical treatment of dermatitis in Kazakhstan, Russia, Belarus, Ukraine, and Uzbekistan. The comparison showed that in all the clinical documents reviewed, topical therapy plays a central role in the treatment of atopic dermatitis, eczema, and other inflammatory skin conditions. The comparative clinical approaches to topical therapy of dermatitis across the selected CIS countries are summarized in Figure 3.

The Republic of Kazakhstan’s clinical protocol for atopic dermatitis states that treatment should be comprehensive and include elimination measures, diet, a hypoallergenic regimen, topical and systemic pharmacotherapy, patient education, and rehabilitation. The goals of therapy are to reduce inflammation and itching, prevent secondary infections, moisturize and soften the skin, restore its protective properties, and improve patients’ quality of life.

The 2024–2026 Russian clinical guidelines emphasize that topical drug therapy is indicated for atopic dermatitis of any severity. The mainstay of treatment consists of emollients, topical glucocorticosteroids, and calcineurin inhibitors, while multicomponent topical preparations containing antibiotics or antifungals are recommended only when a bacterial or fungal infection is confirmed.

The Belarusian clinical protocol for dermatitis and eczema in adults covers a wide range of dermatoses (L20–L30) and identifies topical therapy as a key component of treatment. It specifically lists emollients as agents for restoring the skin barrier, topical corticosteroids, pimecrolimus, moist-to-dry dressings, and—in cases of secondary infection—combination topical preparations containing antibacterial and antifungal components.

The Ukrainian document on the strategy for the treatment and prevention of atopic dermatitis emphasizes the need for systemic and topical treatment, skin care, monitoring of infectious complications, and prevention of flare-ups.

Uzbekistan’s 2025 National Clinical Protocol for Atopic Dermatitis includes topical therapy, proper skin care, systemic pharmacotherapy, physical therapy, education, and prevention; if topical therapy is ineffective, systemic antifungal treatment is permitted.

A detailed comparison of clinical approaches to topical therapy for dermatitis across the CIS countries is presented in Table 9.

To assess the relationship between clinical recommendations and the registered product portfolio, a Clinical–Market Gap Matrix was constructed, combining the results of the comparative analysis of clinical protocols from CIS countries with data from Kazakhstan’s state drug registry. The resulting comparison between clinical recommendations and actual market representation is summarized in Table 10.

## 3. Discussion

### 3.1. Structural Characteristics of the Semi-Solid Dosage Forms Market in the Republic of Kazakhstan

As shown in Table 11, the market is highly concentrated: the top three dosage forms—ointments, gels, and creams—account for 93.8% of all registered products (MCR_3_ = 93.8%), with ointments alone comprising 39.5%.

This indicates limited portfolio diversification and restricted availability of alternative delivery systems such as plasters (2.1%) or transdermal therapeutic systems.

This structural characterization aligns with the findings of marketing studies on the Kazakhstani wound-healing gel market, which also highlighted the need to expand the range of domestically produced drugs and the relevance of developing new wound-healing dosage forms [45].

Moreover, as noted in a review of the current state of chronic wound management in Kazakhstan, statistical data on patients with chronic wounds are neither monitored nor recorded in the country, which creates additional difficulties in assessing the real demand for specialized wound-care products. The authors of this review emphasize the need to establish systematic wound care monitoring and develop an electronic database, as well as the importance of designing and manufacturing new domestic wound-healing products [46].

The absence of systematic statistics on chronic wounds may contribute to underestimation to an underestimation of the real need for wound-healing drugs and, consequently, to the perpetuation of the structural differences in representation identified in this study.

The calculated TMI (56.4%) primarily reflects the relative representation of gels and creams compared to traditional ointments. However, this structural descriptor should not be interpreted as evidence of widespread adoption of innovative delivery platforms, as more complex systems—plasters, transdermal therapeutic systems, hydrogels, bioactive coatings, nanostructured carriers and controlled-release systems—remain limited in the registered portfolio.

Although modern forms (gels, creams, and patches) account for more than half of registered products (TMI = 56.4%), the share of more complex topical systems—hydrogels, bioactive coatings, nanostructured carriers, and controlled-release systems—remains extremely limited, suggesting that the market has transitioned from the absolute dominance of ointments to a broader use of gels and creams but has not yet reached the level of markets where advanced drug delivery platforms play a significant role. The Kazakhstan market cannot yet be regarded as fully technologically advanced in terms of modern wound-care platforms, as the registered portfolio remains predominantly composed of conventional semi-solid dosage forms, unlike the markets of the United States, the European Union, and several Asian countries, where bioactive coatings, intelligent hydrogels, cellular products, and controlled-release systems are actively being introduced.

Thus, the Kazakhstani semi-solid dosage forms market appears to be at an intermediate stage of technological development. It is no longer exclusively “traditional,” since gels and creams account for more than half of the assortment, but it remains less diverse than markets where hydrogels, polymer matrices, bioactive coatings, “smart” dressings, and combination delivery systems play a significant role.

This is particularly relevant for chronic wound care, where efficacy depends not only on the API but also on the base properties—moisture maintenance, controlled release, exudate absorption, and antimicrobial and anti-inflammatory effects. While Kazakhstan has some herbal and topical wound-healing products (oils, ointments, films), modern hydrogels, bioactive dressings, and innovative systems remain underrepresented.

Consequently, the TMI value should be interpreted as a descriptive structural characteristic of the registered portfolio rather than as evidence of technological compliance with international trends. The data reflect a transition from classical ointments to gels and creams, but not yet to high-tech wound-care platforms.

### 3.2. Differences in Therapeutic Representation Across Registered Topical Products

The marked predominance of anti-inflammatory products over wound-healing agents (TGR = 5.6) highlights a structural imbalance in the registered portfolio relative to the multifactorial pathogenesis of chronic wounds (Table 12). 

The limited availability of combination products (6.7%) further restricts multitargeted therapy options (Table 13).

This imbalance is relevant given that chronic wound pathogenesis involves persistent inflammation, microbial contamination, oxidative stress, and impaired angiogenesis [14,15]. Optimal therapy may therefore require comprehensive intervention rather than single-target approaches. The comparison of pathogenetic need with actual market structure (Table 14) illustrates this misalignment.

According to WHO (2025), one in six bacterial infections is antibiotic-resistant, a 40% increase over five years [47]. In wound care, antimicrobial use remains variable and often driven by “fear of infection” rather than protocols, potentially contributing to AMR [48]. The limited representation of combination and multitargeted products (6.7%) may be relevant to the rational use of antibiotics in clinical practice, as patients with chronic wounds may require multiple separate prescriptions instead of a single multifunctional agent.

### 3.3. Comparison with Regional Data: Kazakhstan, Ukraine, Belarus and Russia

For a more accurate interpretation of the results, it is important to compare the Kazakhstani market with data from other countries in the region. The available literature contains individual studies on semi-solid dosage forms and wound-healing agents in Ukraine and Belarus, as well as industry reports on the Russian pharmaceutical market.

The selected countries were chosen because they represent geographically and historically related pharmaceutical markets within the CIS region, sharing broadly comparable healthcare traditions and predominantly generic pharmaceutical portfolios. At the same time, they differ in the degree of regulatory harmonization, reimbursement mechanisms, domestic manufacturing capacity, procurement policies, and the availability of registered medicinal products. These similarities enable meaningful regional comparison, whereas the regulatory and market differences provide important context for interpreting variations in the registered product portfolios. Accordingly, the following comparison is intended to provide regional context for the findings rather than to directly compare healthcare system performance or clinical practice.

The comparison with Ukraine is particularly informative because in the **specialised** segment of drugs for wound care, semi-solid dosage forms accounted for 50.4%, liquid forms for 48.0%, while solid forms constituted 1.6%. Moreover, about 82% of wound-healing products were represented by domestic manufacturers. This contrasts with the situation in Kazakhstan, where wound-healing drugs account for 5.0% of all semi-solid dosage forms, and the market as a whole remains highly import-dependent.

The Belarusian experience is also of interest. A study of the nomenclature of semi-solid dosage forms in the Republic of Belarus showed that soft forms account for about 5% of all registered medicinal products, with complex forms, including plasters and transdermal therapeutic systems, being entirely import-dependent.

In Kazakhstan, the share of semi-solid dosage forms among all registered products is higher—about 16.0%—but the structural pattern is similar: complex delivery systems are limited, and the market is concentrated around classical ointments, gels and creams. A broader regional comparison of semi-solid dosage forms and wound-healing products is presented in Table 15.

The Russian market is a particularly important regional reference point, since Kazakhstan and Russia operate within the close regulatory space of the EAEU, have historically similar pharmaceutical practices and significant medicinal product trade.

According to an industry report by the company Alfarm, the volume of the Russian pharmaceutical market increased by 60.1% between 2019 and 2023, reaching 2.2 trillion roubles in 2023 [52].

The share of Russian manufacturers in monetary terms rose from 30.8% in 2019 to 36.8% in 2023, while in volume terms it reached 62.9% in 2023 compared with 61.3% in 2019. The key comparative indicators are summarized in Table 16.

The Russian market demonstrates a higher level of localisation and greater domestic production potential. This is particularly important in the context of import substitution: if in Kazakhstan, according to published data, only 13.1% of the 7449 registered drugs are domestic, while 86.9% are imported, then in Russia the share of domestic drugs is substantially higher. The Russian report also *emphasises* the growth in the share of domestic manufacturers and the expansion of the drug assortment, indicating a more developed industrial base.

Despite higher localisation, Russia has not fully solved the innovation challenge in complex segments. Nevertheless, its experience shows that state import substitution programmes can reshape the market when backed by investment, R&D, and manufacturing capacity. For Kazakhstan, this implies *prioritising* innovative formulations over generic reproduction to achieve meaningful therapeutic diversification.

Additional perspective for interpreting the obtained results is provided by the analysis of the topical products market in the Central Asian region. According to industry data, Kazakhstan is the largest dermatological products market in Central Asia, where topical forms such as creams and ointments predominate. However, the contract manufacturing market for topical products in Kazakhstan is characterised as “emerging” and import-dependent, with a pronounced structural gap between limited local GMP manufacturing capacity and growing regional demand for specialised dermatological and ophthalmic therapeutic products [52].

This creates both a challenge and an opportunity for Kazakhstan: subject to the development of local manufacturing capacity, the country could become a regional hub for Central Asia and the CIS markets. However, this requires not only investment in production but also the development of proprietary molecules and advanced topical formulations may strengthen the regional pharma-ceutical sector. DHPM-thione represents one possible example of a chemical class that could be explored in future pharmaceutical research.

Thus, the registry-based findings are interpreted not only within the national pharmaceutical market but also in relation to regional therapeutic practice, providing broader context for understanding the organization of topical semi-solid dosage forms in Kazakhstan.

### 3.4. WHO Perspective on Antimicrobial Innovation and Its Relevance to Topical Drug Development

The results of this study can be interpreted not only within the national context of the Kazakhstani pharmaceutical market but also within the broader international context of antimicrobial resistance and the development of new antibacterial agents.

According to the WHO 2025 report, antimicrobial resistance is one of the most serious global health threats, causing more than one million deaths annually; in the absence of intensified measures, up to 39 million deaths directly related to AMR are projected by 2050. The WHO emphasises that as of 15 February 2025, there were 90 antibacterial agents or combinations in clinical development containing at least one new therapeutic entity—50 traditional antibacterial agents and 40 non-traditional agents—and notes that even taking into account drugs in development and those approved in the last seven years, the existing antibacterial pipeline remains insufficient to counter the rise in drug-resistant infections. The broader international context is summarized in Table 17.

This is particularly important for two reasons. First, infected wounds, diabetic ulcers, burns, and chronic skin injuries are often complicated by bacterial contamination and biofilm formation. Second, topical antibacterial use should be considered within the context of antimicrobial stewardship and rational antibiotic therapy. The predominance of classical antibacterial agents in the market, without sufficient multifunctional alternatives capable of reducing inflammation and supporting regeneration, may increase dependence on traditional antibiotics.

The WHO defines antibacterial innovativeness primarily as the absence of known cross-resistance, with additional indicators being a new chemical class, new molecular target, or new mechanism of action. DHPM-thione satisfies one criterion—it belongs to a chemical class absent from the **analysed** registry.

According to WHO (2025), the antibacterial pipeline has declined from 97 agents in 2023 to 90 in 2025. Of these, only 15 qualify as innovative, and only 5 are effective against WHO critical-priority pathogens. Since 2017, only two new chemical classes have reached marketing authorisation. While the preclinical pipeline remains active (232 programmes), 90% of developing companies have fewer than 50 employees, highlighting R&D ecosystem fragility [47].

Against this backdrop, the development of new chemical classes for topical application becomes particularly significant. DHPM-thione, a member of a chemical class not represented in the **analysed** registry, has demonstrated antioxidant and anti-inflammatory activity in preclinical studies and may therefore warrant further investigation as a candidate for topical pharmaceutical development.

In this context, the development of innovative domestic molecules, such as DHPM-thione, can become one of the elements in building a full-fledged, self-sufficient pharmaceutical ecosystem.

### 3.5. WHO Diagnostic Perspective and Topical Drug Development

The WHO (2025) emphasises that combating AMR requires not only new drugs but also improved diagnostics—tests for distinguishing bacterial from non-bacterial infections, pathogen identification, and antimicrobial susceptibility testing. Diagnostic systems should support rational antibiotic prescribing and reduce inappropriate use [47].

This has direct relevance to wound care. In clinical practice, chronic and inflammatory skin lesions are often treated empirically, particularly at primary care level, without microbiological confirmation. Thus, expanding the antibacterial segment alone does not fully address the problem. A complementary approach discussed in the literature is the development of multitargeted drugs that simultaneously reduce inflammation, control microbial contamination, decrease oxidative stress, and support tissue regeneration.

Critical diagnostic gaps persist globally: limited access to biomarker tests (e.g., C-reactive protein, procalcitonin) and simple diagnostic tools at primary healthcare level, disproportionately affecting low-resource countries [47]. These observations are relevant to the Kazakhstani registry data: even with an expanded range of antibacterial soft forms, their use without rapid and accessible diagnostics may remain empirical, risking irrational antibiotic use and resistance development. The key implications are summarized in Table 18.

In this broader context, multitargeted compounds such as DHPM-thione may warrant further investigation as potential complements to existing therapeutic approaches.

Thus, the study results may be interpreted not as a call for a simple increase in the number of local antibiotics, but as an argument in favour of developing more comprehensive topical agents. In the context of rising AMR and the limited antibacterial pipeline, future topical formulations with multitargeted properties may have the potential to complement existing therapeutic approaches, where possible, reduce the need for systemic antibiotic therapy, maintain local inflammation control and accelerate tissue repair. It is within this logic that DHPM-thione may be considered as a potential class for further pharmaceutical research.

### 3.6. Representation of Heterocyclic Compounds: The Absence of DHPM-Thione from the Registry

The structural-chemical analysis showed that the Kazakhstani semi-solid dosage forms market already contains a significant number of heterocyclic active pharmaceutical ingredients. A total of 490 drugs with heterocyclic structures were identified, representing 55.0% of all *analysed* soft forms. Among these, imidazole derivatives were represented by 112 drugs, or 12.6% of the total soft forms; triazoles by 58 drugs, or 6.5%; purines by 42 drugs, or 4.7%; pyridines by 35 drugs, or 3.9%; and pyrimidines by 18 drugs, or 2.0%.

However, dihydropyrimidine and dihydropyrimidine-2(1H)-thione derivatives were entirely absent. The corresponding distribution is summarized in Table 19.

The key observation is that the market already contains heterocyclic compounds, particularly antifungal imidazoles and triazoles. Therefore, the absence of DHPM-thione cannot be explained by the low applicability of heterocycles in soft forms. Rather, it reflects the absence of translation of a particular chemical class into registered topical drugs.

This observation is particularly noteworthy because DHPM and DHPM-thione are described in the literature as compounds with a broad spectrum of biological activity. The literature review notes that DHPM/thiones possess anti-inflammatory, antibacterial, antioxidant and other pharmacological potential; at the same time, a systematic review of DHPM showed that most data remain in vitro, and only 12 of 115 studies included in vivo experiments. This is important for correct interpretation: DHPM-thione should not be presented as an already proven clinical drug for wounds, but it can be reasonably positioned as a promising preclinical class for further research.

Of particular interest is the contemporary direction of DHPM-containing nanoplatforms for infected diabetic wounds. The literature review indicates that a bacterium-sensitive nanoplatform with DHPM fragments promoted biofilm dispersal, ROS scavenging, inflammation reduction, wound transition to the proliferative phase, enhanced angiogenesis and granulation tissue formation. This work illustrates the potential relevance of DHPM chemistry to wound-care research, although it remains at the experimental nanoplatform stage rather than registered ointments, gels or creams.

Scientific interest in dihydropyrimidinones and their thione analogues has grown considerably in recent years. A review published in 2025 emphasises that the Biginelli reaction remains a versatile method for obtaining 3,4-dihydropyrimidin-2(1H)-ones, 3,4-dihydropyrimidin-2(1H)-thiones and related heterocycles, which attract significant attention due to their pharmacological properties and diverse biological activities. These compounds demonstrate activity against cancer, Alzheimer’s disease, microbial infections, tuberculosis, inflammation, malaria, filariasis, hypertension, and also possess antioxidant and antiviral properties [53,54]. Another review confirms that dihydropyrimidinones play a critical role as anticancer, antioxidant, antimicrobial, anti-inflammatory and antihypertensive agents [55,56,57].

Of particular note is the study of a bacterium-sensitive nanoplatform with DHPM fragments for the treatment of infected diabetic wounds, published in Acta Biomaterialia (2025). This work showed that DHPM fragments of the nanoplatform act as reactive oxygen species (ROS) scavengers, allowing the reduction in oxidative stress and inflammatory responses, facilitating the transition of the healing process to the proliferative phase, characterised by enhanced extracellular matrix deposition, angiogenesis and granulation tissue formation [53]. Thus, DHPM-containing systems demonstrate the ability to simultaneously address several key problems of chronic wounds: persistent inflammation, oxidative stress and impaired regeneration.

These data create a foundation for further research into semi-solid dosage forms based on DHPM-thione. The absence of this class from the State Register of Kazakhstan, together with available preclinical evidence, suggests a potential area for further research.

### 3.7. DHPM-Thione as a Potential Subject for Future Research

From a research perspective, the findings of the present study provide a framework for considering DHPM-thione as a candidate chemical class for future investigation in multitargeted topical dosage forms.The market structure identified in the present study provides useful context for prioritizing future pharmaceutical research. Anti-inflammatory agents constitute the largest pharmacotherapeutic category (28.0% of registered products), whereas wound-healing agents (5.0%) and combination products (6.7%) are represented less frequently within the registered portfolio.

In addition, no medicinal products containing dihydropyrimidine-2(1H)-thione (DHPM-thione) derivatives were identified in the national medicines registry. These observations describe structural characteristics of the current product portfolio rather than evidence of unmet clinical need or commercial potential. Nevertheless, when interpreted alongside the published scientific literature, they illustrate how registry-based market analysis can be integrated with structural–chemical evidence to identify research hypotheses for future pharmaceutical development.

A recent systematic review of 60 studies demonstrated that DHPM-thione derivatives have been extensively investigated in preclinical models across multiple therapeutic areas. Attempting to position DHPM-thione solely as a new anti-inflammatory agent would place it in a mature and crowded segment.

An alternative approach would be to consider DHPM-thione as a chemical class for multitargeted dosage forms, potentially addressing several identified areas of limited representation. Its comparative positioning relative to these market segments is presented in Table 20:

i. wound-healing drugs—5.0% of the market;
ii. combination products—6.7% of the market;
iii. the absence of DHPM-thione—0%.

**Figure 4:**
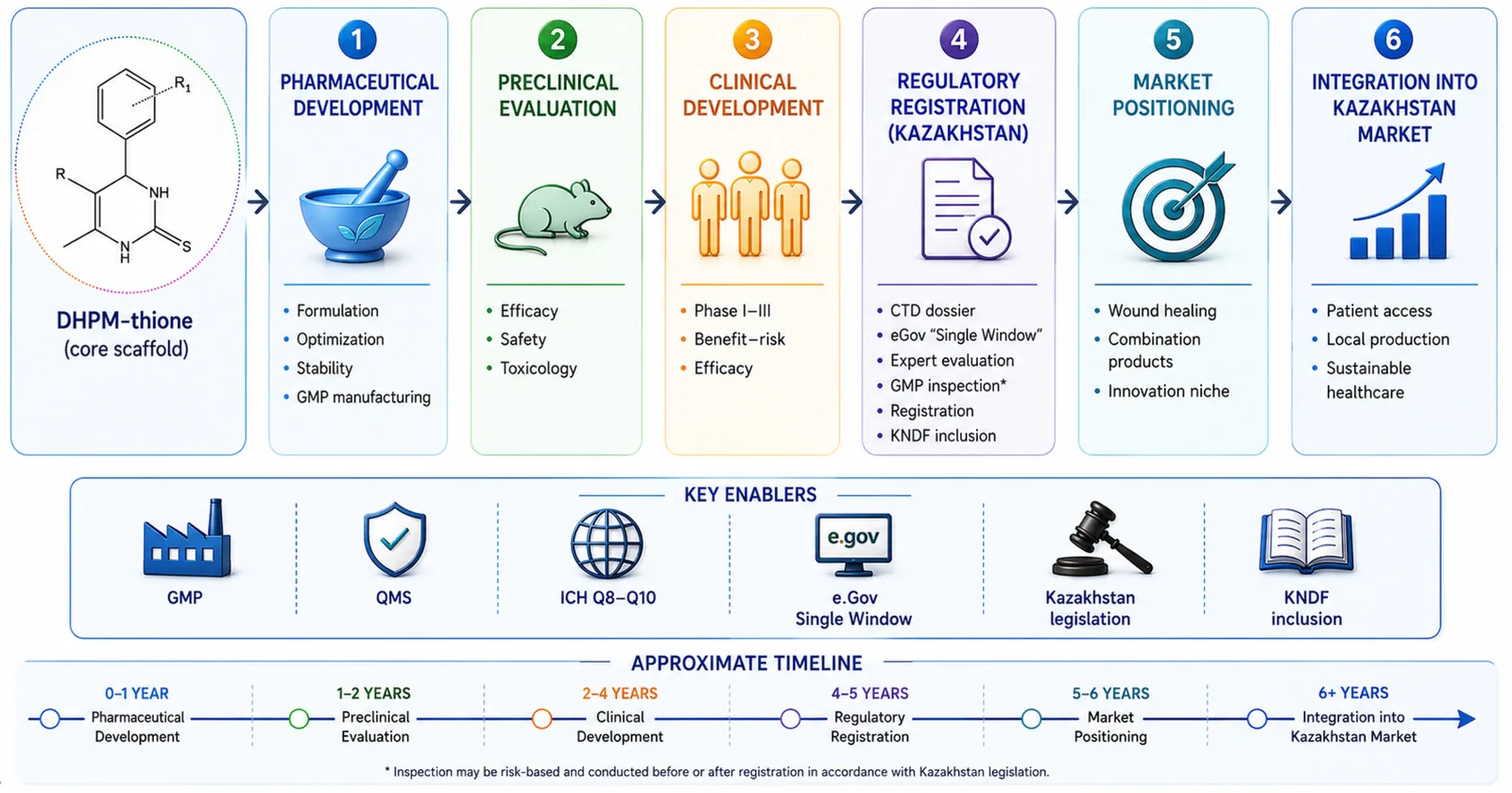
Conceptual roadmap for the development and positioning of DHPM-thione-based topical medicinal products in Kazakhstan. The diagram was prepared using ChatGPT Plus 5.5 (OpenAI) solely for visualization of the summarized data.

Global trends confirm increasing interest in advanced topical delivery systems incorporating nanocarriers, liposomes, and polymer systems [56], with the advanced drug delivery market projected to reach $2.9 trillion by 2032 [57,58,59,60]. The proposed development pathway is illustrated in Figure 4.

From a regulatory perspective, the development of DHPM-thione-based topical products in Kazakhstan would operate under the evolving framework of the Eurasian Economic Union (EAEU), with a transition period ending in 2026. The primary competent authorities are the Committee for Medical and Pharmaceutical Control (CMPC), responsible for state registration, and the National Center for Expertise of Medicines and Medical Devices (NCE), which conducts safety, quality, and efficacy evaluations [39,40,41]. However, unlike the FDA and EMA, Kazakhstan currently lacks specialized guidance documents for evaluating the equivalence of complex topical products—including in vitro release testing (IVRT), in vitro permeation testing (IVPT), and microstructural (Q3) equivalence. The FDA recommends IVRT with r^2^ ≥ 0.97, while EMA criteria are less stringent (r^2^ > 0.90), and EMA accepts tape stripping studies for local bioavailability assessment, whereas FDA does not [60,61,62]. This regulatory gap is compounded by the inherent batch-to-batch variability of reference products, which may complicate equivalence demonstration even when guidelines exist [63].

Based on the present market analysis and the current regulatory framework in the Republic of Kazakhstan, a conceptual roadmap for the development and positioning of DHPM-thione-based topical medicinal products is proposed (Figure 4). The roadmap summarizes the sequential stages from pharmacological validation and pharmaceutical development under GMP principles to preclinical and clinical evaluation, regulatory registration through the national “Single Window” procedure, and potential market introduction. It is intended to illustrate a possible development pathway derived from the identified market structure and current regulatory requirements rather than to predict future clinical or commercial outcomes. The roadmap also highlights the need for Kazakhstan to either develop local pharmacopoeial methods or adapt EAEU rules to address the specific complexities of topical semi-solid products, ensuring that future innovations can be evaluated through robust, internationally aligned regulatory pathways.

### 3.8. Limitations of the Study

This study has several limitations that should be considered when interpreting the findings.

First, the analysis was based exclusively on registration data from the State Register of Medicinal Products of the Republic of Kazakhstan. Consequently, the results reflect the structure and diversity of the registered pharmaceutical market rather than actual product availability, sales volumes, prescribing patterns, or clinical utilization. Some registered products may not be actively marketed or available in pharmacies.

Second, the study does not include information on market sales, consumption patterns, or healthcare utilization, which would be necessary to evaluate the actual demand for specific therapeutic categories and their economic significance.

Third, the classification of drugs into pharmacotherapeutic categories was based on the primary therapeutic indication recorded in the national medicines register. Some products may possess additional therapeutic properties that are not reflected in the primary classification. Likewise, the structural classification of APIs was based on the predominant heterocyclic scaffold, although certain molecules contain multiple heterocyclic fragments.

Fourth, the absence of DHPM-thione-containing products in the national medicines registry should not be interpreted as evidence of an unmet clinical need, lack of therapeutic potential, or commercial feasibility. Rather, it indicates that this chemical class is currently not represented among registered medicinal products in Kazakhstan. The discussion of DHPM-thione derivatives is based on published preclinical evidence and is intended solely to illustrate their potential relevance for future pharmaceutical research.

Fifth, the present study is descriptive in nature and does not include hypothesis testing, clinical outcome assessment, pharmacoeconomic evaluation, or comparative effectiveness analyses. Therefore, no conclusions regarding clinical efficacy, safety, cost-effectiveness, or patient outcomes can be drawn from the presented data.

Sixth, the study does not include Kazakhstan-specific epidemiological data on the prevalence of chronic wounds, diabetic foot ulcers, or inflammatory skin diseases. As reported previously, systematic nationwide monitoring and registration of patients with chronic wounds are currently unavailable in Kazakhstan.

Consequently, it is not possible to directly assess the relationship between the observed market structure and the actual burden of these conditions. Therefore, the observed distribution of therapeutic categories should be interpreted as a structural characteristic of the registered medicines portfolio rather than direct evidence of unmet clinical demand.

Finally, the comparison with clinical guidelines from selected CIS countries was performed solely to provide contextual interpretation of the observed market structure and should not be regarded as an assessment of clinical practice, prescribing behaviour, or therapeutic appropriateness. The reviewed guidelines represent recommendations, whereas actual clinical practice may differ.

## 4. Materials and Methods

### 4.1. Data Source

This study was designed as a cross-sectional descriptive pharmaceutical market analysis of topical semi-solid dosage forms registered in the Republic of Kazakhstan. The study followed the principles of descriptive registry-based research and was aimed at characterizing the current structure of the national pharmaceutical market without assessing the clinical effectiveness, safety, or comparative therapeutic value of medicinal products.

The national medicines registry provides a comprehensive regulatory description of the pharmaceutical market, including the composition of registered medicinal products, dosage forms, manufacturers, countries of origin, and active pharmaceutical ingredients. Accordingly, the present analytical framework was designed to characterize the structural organization of the registered pharmaceutical portfolio. The analysis therefore focuses on structural attributes of the market, whereas dimensions such as sales volume, prescribing patterns, healthcare utilization, pricing, and pharmacoeconomic outcomes require different data sources and were beyond the objectives of the present registry-based investigation.

The primary data source was the State Register of Medicinal Products of the Republic of Kazakhstan maintained by the National *Center* for Expertise of Medicines and Medical Devices under the Ministry of Health of the Republic of Kazakhstan. The registry contains publicly available information on registered medicinal products, including trade names, international nonproprietary names (INNs), dosage forms, active pharmaceutical ingredients (APIs), Anatomical Therapeutic Chemical (ATC) codes, manufacturers, countries of manufacture, marketing authorization holders, and registration status.

In addition, summary statistics from the State Register of Medicines of the Republic of Kazakhstan as of 13 June 2026 were used, including the number of valid registration certificates, the number of medicines registered under the national procedure and in accordance with EAEU regulations, as well as information on domestically produced medicines [39,40,41].

### 4.2. Inclusion and Exclusion Criteria

The analysis included medications classified as soft and topical dosage forms. The study included topical semi-solid dosage forms indicated for the treatment of wounds, inflammatory skin diseases, burns, dermatitis, fungal infections, bacterial skin infections, and related conditions, according to the approved indications provided in the State Register. The analysis included only medicinal products with active marketing authorization at the time the registry data were extracted. Expired or inactive registration records were not considered.

Products with multiple approved therapeutic indications were not excluded from the analysis. Each product was assigned to a single pharmacotherapeutic category based on its primary therapeutic indication, determined using the Anatomical Therapeutic Chemical (ATC) classification, the international nonproprietary name (INN), and the official prescribing information recorded in the State Register of Medicinal Products of the Republic of Kazakhstan. Combination products containing active ingredients from different therapeutic groups were classified separately as combination products and were not counted in other categories. This approach ensured that each registration record contributed only once to the analysis and prevented double counting. Systemic dosage forms, medical devices, veterinary drugs, as well as technical records and duplicate entries were excluded from the initial dataset, as summarised in Table 21.

### 4.3. Pharmacotherapeutic Classification

To ensure the reproducibility of the study, all drugs were classified into pharmacotherapeutic groups based on the ATC classification (Anatomical Therapeutic Chemical Classification System), international nonproprietary names, prescribing information, and primary therapeutic indication [64,65]. The classification scheme applied is shown in Table 22.

After classification, the absolute number of registration records, their percentage of the total dataset, and their distribution by country of manufacture were calculated for each pharmacotherapeutic group. Quantitative findings derived from this classification are reported in the Section 2.

### 4.4. Structural and Chemical Classification of Active Pharmaceutical Ingredients

**Figure 5:**
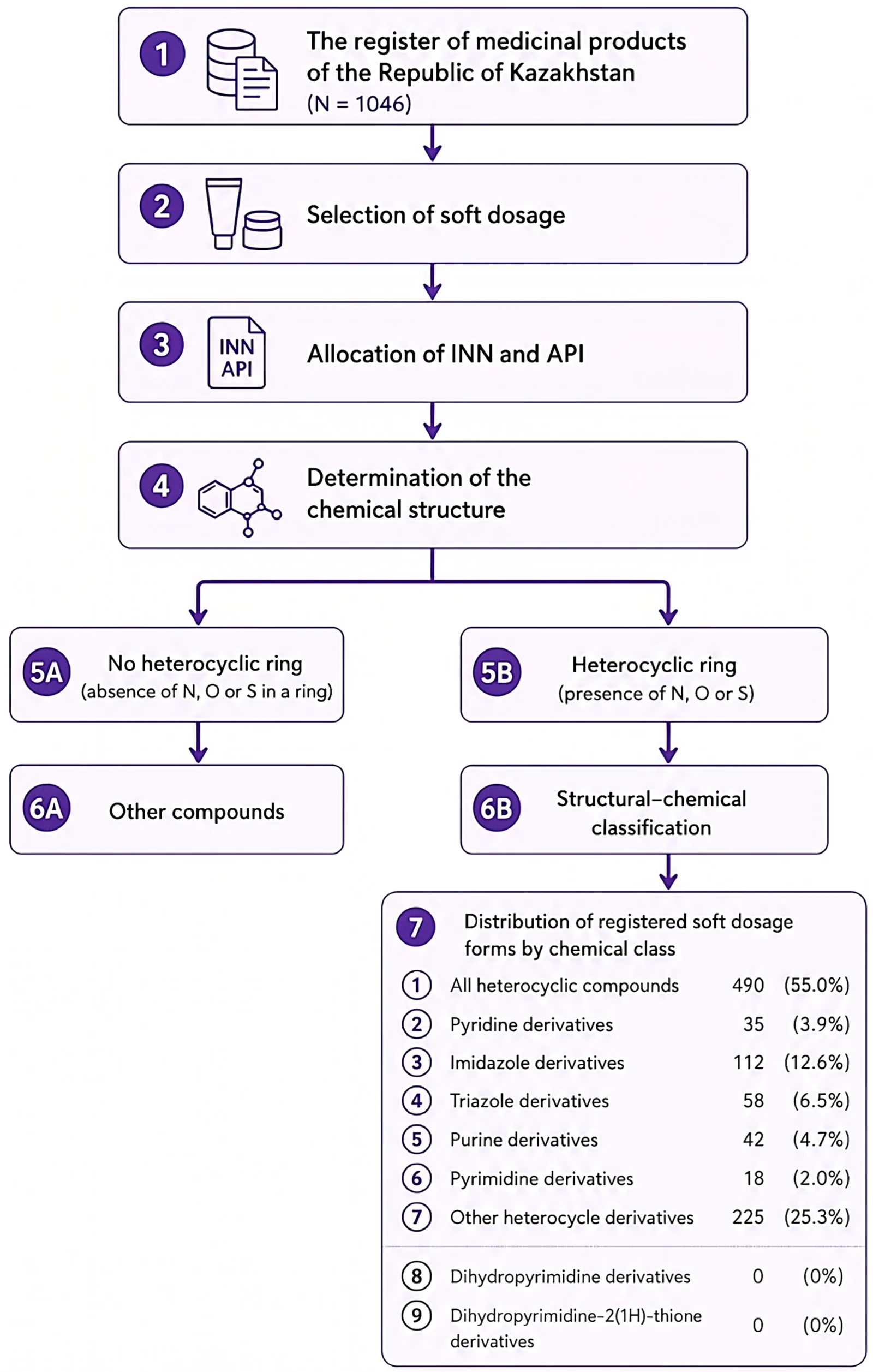
Flowchart of the structural-chemical classification of active pharmaceutical ingredients in semi-solid dosage forms registered in the Republic of Kazakhstan.

To characterize the chemical diversity of the market for semi-solid dosage forms, a structural-chemical classification of active pharmaceutical ingredients (APIs) was conducted. The analysis was carried out sequentially in several stages. The workflow used for the structural-chemical classification of APIs is presented in Figure 5.

In the first stage, the International Nonproprietary Name (INN) and active pharmaceutical ingredient (API) were determined for each drug based on data from the State Register of Medicines of the Republic of Kazakhstan [66].

In the second stage, the chemical structure of each API was determined using open-access chemical databases (PubChem, ChEBI, DrugBank, ChemSpider) and official pharmacopoeial sources [65].

In the third stage, a structural analysis of the molecules was conducted to identify the presence of heterocyclic fragments. A compound was classified as heterocyclic if its structure contained at least one cyclic fragment containing nitrogen, oxygen, or sulfur atoms [67,68].

The structural-chemical classification criteria for active pharmaceutical ingredients are summarised in Table 23. In the fourth stage, all heterocyclic compounds were classified into chemical classes based on their dominant structural core. Compounds containing multiple heterocyclic fragments were assigned to the class corresponding to their predominant structural scaffold.

### 4.5. Analysis of the Structure of the Semi-Solid Dosage Forms Market

To quantitatively characterize the market for semi-solid dosage forms in the Republic of Kazakhstan, an analysis of the product mix of registered drugs was conducted using descriptive statistical indicators that allow for an assessment of the distribution of dosage forms and the degree of market concentration [66,67,68,69].

In the first stage, the absolute number of registered drugs for each dosage form (*N_i_*) and their share in the overall structure of the market for semi-solid dosage forms were determined. The relative share of each dosage form (*s_i_*) was calculated using the formula:

(1) $$s_{i}=\frac{N_{i}}{N_{total}}\times 100$$

where *N_i_*—the number of drugs in the appropriate dosage form, *N_total_*—the total number of semi-solid dosage forms included in the study.

To assess the degree of dominance of individual dosage forms, the market concentration ratio for the top three categories (Market Concentration Ratio, MCR_3_) was calculated:

(2) $$MCR_{3}=\frac{N_{1}+N_{2}+N_{3}}{N_{total}}\times 100\;$$

where *N*_(1)_, *N*_(2)_ and *N*_(3)_ correspond to the number of drugs in the three most common dosage forms.

MCR_3_ values below 45% were interpreted as indicating low market concentration, 45–60% as moderate, 61–75% as high, and over 75% as very high concentration. This widely used indicator was employed to describe the degree of concentration among dosage-form categories [70,71].

To further characterize market diversity, the Shannon Diversity Index (*H*) was calculated:

(3) $$H=-\sum_{i=1}^{k}p_{i}\ln\left(pi\right)$$

where *p_i_* represents the share of the relevant dosage form in the overall market structure, and *k* is the number of categories analyzed.

The Shannon index is a standard measure of diversity originally developed in ecology but widely applied in economics and market research to assess the uniformity of distribution across categories [72,73]. In this study, the Shannon index was adapted to assess the diversity of dosage form categories. This adaptation is valid because the index reflects the uniformity of distribution of objects across categories, regardless of the nature of the units being analyzed. A higher H value indicates a more balanced distribution across dosage forms, while lower values indicate concentration in fewer categories.

To assess the level of technological development in the market, all dosage forms were divided into two categories. Traditional dosage forms included ointments, pastes, and liniments, characterized by a long history of use and relatively simple manufacturing technologies. Modern dosage forms included gels, creams, hydrogels, patches, and transdermal therapeutic systems, which involve the use of modern polymeric carriers, modified-release technologies, and improved drug delivery systems.

The proportions of traditional (*TS*) and modern (*MS*) dosage forms were calculated using the following formulas:

(4) $$TS=\frac{N_{Trad}}{N_{total}}\times 100$$

(5) $$MS=\frac{N_{Mod}}{N_{total}}\times 100\;$$

where *N_Trad_*—number of traditional dosage forms, a *N_Mod_*—number of modern dosage forms.

The Technological Maturity Index (*TMI*) was used as a descriptive structural indicator reflecting the relative proportion of dosage forms that were operationally classified as modern or traditional for the purposes of market characterization. The classification was introduced solely to describe the structural composition of the registry dataset and should not be interpreted as a validated measure of technological innovation, pharmaceutical advancement, product quality, or clinical superiority. The TMI was calculated as the share of modern dosage forms in the total market volume:

(6) $$TM\;I=\frac{N_{Mod}}{N_{total}}\times 100$$

Higher *TMI* values indicate a larger proportion of dosage forms classified as modern according to the operational classification adopted in this study. The index was selected because it allows for straightforward calculation and comparison with other markets as a descriptive indicator.

To compare the prevalence of modern and traditional dosage forms, the Form Innovation Ratio (*FIR*) was calculated:

(7) $$FIR=\frac{N_{Mod}\;}{N_{Trad}\;}\;$$

*FIR* values above 1 indicate a predominance of modern dosage forms; a value of 1 indicates equal representation; and values below 1 reflect the dominance of traditional technologies. This ratio complements the *TMI* by providing a relative measure of modern versus traditional forms.

MCR_3_, the Shannon index, *TMI*, and *FIR* were used to characterize market structure, portfolio diversification, and technological profile. These indices are descriptive analytical tools for summarizing registry data and should not be interpreted as validated measures of clinical need, therapeutic efficacy, or market performance.

### 4.6. Gap Analysis of Therapeutic Categories

To identify differences in representation among the main therapeutic categories of the market for semi-solid dosage forms, a comparative analysis was conducted. The comparison was informed by the multifactorial nature of wound management: effective treatment of chronic and infected wounds requires a comprehensive approach targeting three key pathophysiological mechanisms: suppression of inflammation, control of infection, and stimulation of tissue regeneration processes [74,75,76,77,78].

In the first stage, for each pharmacotherapeutic category, we determined the absolute number of drugs (*N_j_*) and their share in the overall market structure of semi-solid dosage forms (*S_j_*):

(8) $${\;S}_{j}=\frac{N_{j}}{N_{total}}\times 100\;$$

where *N_j_*—the number of drugs in the relevant category; *N_total_*—the total number of semi-solid dosage forms included in the study.

The analysis included four main categories relevant to wound care and inflammatory skin conditions:

i. wound-healing and reparative agents;
ii. antibacterial and antimicrobial agents;
iii. anti-inflammatory agents;
iv. combination agents.

Classification was based on ATC codes, international nonproprietary names, and the primary therapeutic indication of the drug.

To quantitatively assess the difference in representation between individual categories, the Therapeutic Gap Ratio (*TGR*) was calculated:

(9) $$TGR_{A\to B}=\frac{S_{A}}{S_{B}}\;$$

where *S_A_* and *S_B_* represent the shares of the categories being compared.

A *TGR* value of 1 indicates equal representation of the categories. Values greater than 1 indicate a predominance of category A, while values less than 1 indicate a predominance of category B. Of particular interest were the coefficients:

$$TGR_{AntiInfl\to WoundHeal}$$

and

$$TGR_{AntiBact\to WoundHeal},$$

Since they allow for an assessment of the relative representation of wound-healing agents compared to anti-inflammatory and antibacterial agents.

The *TGR* is a simple ratio-based measure that was selected because it provides an intuitive and transparent quantification of differences in representation between therapeutic categories. Unlike more complex indices, the TGR does not require subjective weighting or assumptions about optimal market structure; it simply expresses how many times more frequently one category is represented in the registry compared to another. The results of the analysis were presented in the form of a distribution of therapeutic categories and comparative ratios, allowing for the identification of categories with lower or higher representation in the registered product portfolio.

### 4.7. Statistical Data Analysis and Visualization

Statistical analysis of the data was performed after preliminary cleaning of the registry to remove empty rows, technical records, duplicate headings, and irrelevant dosage forms. The unit of analysis was an individual registration record for a drug.

Quantitative indicators were presented as absolute values and relative percentages. For categorical variables, distribution frequencies were calculated by dosage form, pharmacotherapeutic group, country of manufacture, manufacturer, and chemical class of active pharmaceutical ingredients.

The following indicators were used to analyze the market structure. The descriptive indicators used to characterize the market structure are summarized in Table 24.

The results were visualized using tables, bar charts, and pie charts.

To provide a more comprehensive quantitative characterization of the registered pharmaceutical portfolio, the analysis combined established structural measures (MCR_3_ and the Shannon diversity index) with complementary indicators describing different dimensions of the registry dataset. MCR_3_ was used to quantify concentration of dosage-form categories, whereas the Shannon index characterized structural diversity. TMI and FIR summarized the technological composition of the registered dosage forms according to the predefined classification adopted in this study, while TGR quantified differences in the representation of the principal therapeutic categories. Collectively, these indicators describe complementary structural characteristics of the registered pharmaceutical market and support systematic comparison of dosage-form, technological, and therapeutic distributions within the registry. For the subsequent interpretation of the identified market structure, the analytical framework was additionally informed by literature addressing pharmaceutical innovation and R&D productivity [77,78,79], established approaches to product portfolio and BCG matrix analysis [80,81,82], scenario-based forecasting and pharmaceutical foresight [83,84], and pharmacoeconomic, cost-avoidance, and cost-effectiveness principles [83,84,85]. These sources were used to provide methodological context for the subsequent strategic interpretation of the descriptive registry findings rather than to modify the underlying registry-derived indicators.

All calculations were performed using descriptive statistical methods, as the study was a cross-sectional descriptive analysis of a drug registry and statistical methods for testing clinical hypotheses were not applied. This approach is consistent with the design of cross-sectional descriptive studies based on registry data, where the primary objective is to provide a quantitative description of the market structure [67,68]. Accordingly, the analysis was limited to quantitative description of the registry structure and comparison of representation across dosage forms, therapeutic categories, and chemical classes.

## 5. Conclusions

This study provides a comprehensive descriptive analysis of the semi-solid dosage forms market in the Republic of Kazakhstan based on data from the State Register of Medicinal Products as of June 2026. The analysis establishes a reference dataset describing the current pharmaceutical landscape, including dosage forms, therapeutic categories, manufacturers, and API chemical classes.

The market for topical semi-solid dosage forms in Kazakhstan comprises 1046 registered products, representing approximately 16.0% of all registered medicinal products. The market is characterized by a high degree of concentration around three dosage forms: ointments (39.5%), gels (28.9%), and creams (25.4%), which together account for 93.8% of all registered semi-solid dosage forms (MCR_3_ = 93.8%). The Shannon Diversity Index of 1.26 confirms this concentration, indicating that while six dosage form categories are present, the distribution is uneven. Gels and creams accounted for 56.4% of registered products (TMI), while traditional forms (ointments, liniments, pastes) accounted for 43.6%. However, the share of more complex delivery systems—plasters, hydrogels, and transdermal systems—remains limited.

Analysis of the pharmacotherapeutic structure revealed differences in representation among therapeutic categories. Anti-inflammatory products (28.0%) and antibacterial drugs (13.5%) were more frequently represented than wound-healing agents (5.0%) and combination products (6.7%). The Therapeutic Gap Ratio of 5.6 between anti-inflammatory and wound-healing products indicates that anti-inflammatory drugs were represented 5.6 times more frequently than wound-healing agents in the registered portfolio. These observations suggest that the registered product portfolio shows higher representation of products targeting inflammation and infection control compared to products specifically indicated for tissue regeneration.

Heterocyclic compounds represent 55.0% of registered APIs, with imidazoles (12.6%), triazoles (6.5%), and pyrimidines (2.0%) being the most represented classes, while no DHPM-thione derivatives were identified. The market is predominantly supplied by foreign manufacturers, with Russian products (35–40%) and EU manufacturers (20–25%) leading, while domestic producers account for 15–20% of registrations, primarily in traditional dosage forms.

Comparison with CIS clinical guidelines showed that while topical anti-inflammatory therapy is well represented, emollients, barrier-restoring agents, and combination products are less frequently represented, suggesting a discrepancy between the registered portfolio and regional clinical recommendations. This aligns with global AMR concerns [69]; WHO emphasizes that the antibacterial pipeline is insufficient and multitargeted approaches warrant further investigation.

The absence of DHPM-thione from the registry, combined with preclinical evidence of its biological activity, suggests a potential area for further research. However, this study is descriptive and does not provide evidence on clinical effectiveness, safety, or commercial viability. Further research—formulation development, preclinical evaluation, and clinical studies—would be required.

From a regulatory perspective, topical product registration in Kazakhstan operates under EAEU rules, with transition ending in 2026. The competent authorities are CMPC and NCE. Unlike the FDA and EMA, Kazakhstan lacks specialized guidance for evaluating complex topical products—including IVRT and Q3 equivalence. This gap, compounded by batch-to-batch variability of reference products, challenges generic development. Addressing it through EAEU adaptation or local pharmacopoeial methods could support future innovations, including potential DHPM-thione-based products.

In summary, this study provides the first comprehensive assessment of the registered topical semi-solid dosage forms market in Kazakhstan. The absence of DHPM-thione-containing products supports consideration of this chemical class for future research, though no conclusions on clinical effectiveness or commercial potential can be drawn.

## Figures and Tables

**Table 1:** Structure of the semi-solid dosage forms market in the Republic of Kazakhstan.

Dosage Form	n	%
Ointments	413	39.5
Gels	302	28.9
Creams	266	25.4
Liniments	26	2.5
Plasters	22	2.1
Pastes	17	1.6
**Total**	**1046**	**100**

**Note**: The three most prevalent dosage forms—ointments, gels and creams—collectively accounted for 93.8% of all semi-solid dosage forms (MCR_3_ = 93.8%), indicating a very high level of market concentration.

**Table 2:** Geographical structure of manufacturers of semi-solid dosage forms.

Region	Number of Registrations (n)	Share of Total Soft Forms (%)
Russia	375	35.9
European Union	225	21.5
Kazakhstan	175	16.8
India	125	12.0
Turkey	40	3.8
Republic of Korea + Japan	30	2.8
Other countries	76	7.1
**Total**	**1046**	**100**

**Note:** Values are normalized based on the distribution ranges reported in pharmaceutical market studies [40,41]. The calculation follows a two-step procedure: (1) midpoints of reported percentage ranges were calculated for each region; (2) the sum of midpoints (1092) was normalized to the total number of registered semi-solid dosage forms (*N* = 1046).

**Table 3:** Market structure by origin of active pharmaceutical ingredients.

API Category	Number of Drugs (n)	Share of Total Soft Forms (%)
Synthetic compounds	771	73.7
Semi-synthetic compounds	137	13.1
Natural compounds	95	9.1
Biological drugs	43	4.1
DHPM/DHPM-thione	0	0
**Total**	**1046**	**100**

**Table 4:** Estimated structure of the domestic segment of the semi-solid dosage forms market.

Manufacturer	Estimated Share Among Domestic Manufacturers (%)	Estimated Registrations (n)	Main Products
Khimpharm (SANTO)	34.2	~60	Gels, ointments, topical forms
Nobel Almaty	15.8	~28	Creams and gels
DOSFARM	13.2	~23	Topical dosage forms
Farmatsiya 2010	7.9	~14	Ointments
Other manufacturers	28.9	~50	Fragmented assortment (Kyzylmay, Pharm Aktobe, Pharmacy 2010, Shansharov-Pharm, KFK LLP, and others)
**Total**	**100**	**~175**	

**Note:** Estimates are based on an approximate domestic market share of 16.7% (175 of 1046 registered semi-solid dosage forms). Values represent normalized estimates derived from available registry data and published market analyses. According to the registry data, domestic manufacturers are predominantly represented by traditional dosage forms (ointments, gels, and creams), whereas more technologically complex delivery systems are primarily represented by imported products [41].

**Table 5:** Indicators describing the structural composition of dosage forms.

I93.	Value
MCR_3_	93.8%
Shannon Index (*H*)	1.26
TMI (Technological Maturity Index)	56.4%
Traditional dosage forms (ointments, liniments, pastes)	43.6%

**Table 6:** Main therapeutic categories of semi-solid dosage forms.

Category	Number of Drugs	Share (%)
Anti-inflammatory	293	28.0
Antibacterial	141	13.5
Combination products	70	6.7
Wound-healing	52	5.0

**Note:** The table presents the four main therapeutic categories relevant to wound care and inflammatory skin conditions. Percentages are calculated relative to the total number of registered semi-solid dosage forms (*N* = 1046). Other pharmacotherapeutic categories (antifungals, antiseptics, corticosteroids, etc.) are not shown in this summary table.

**Table 7:** Therapeutic representation ratios between the main pharmacotherapeutic categories.

Comparison	TGR
Anti-inflammatory/Wound-healing	5.6
Antibacterial/Wound-healing	2.7
Anti-inflammatory/Combination	4.18
Antibacterial/Combination	2.01
Combination/Wound-healing	1.34

**Table 8:** Distribution of registered semi-solid dosage forms by chemical class.

Compound Class	Number of Drugs, n	Share of Total Soft Forms, %
All heterocyclic compounds	490	55.0
Pyridine derivatives ^1^	35	3.9
Imidazole derivatives ^2^	112	12.6
Triazole derivatives ^3^	58	6.5
Purine derivatives ^4^	42	4.7
Pyrimidine derivatives ^5^	18	2.0
Other heterocycle derivatives ^6^	225	25.3
Dihydropyrimidine derivatives	0	0
Dihydropyrimidine-2(1H)-thione derivatives	0	0

**Note:** ^1^ Pyridine derivatives—represented exclusively by piroxicam (a non-steroidal anti-inflammatory drug containing a pyridine ring). ^2^ Imidazole derivatives—metronidazole, clotrimazole, ketoconazole, miconazole, econazole, sertaconazole, bifonazole and others. ^3^ Triazole derivatives—fluconazole, terconazole and other antifungal agents. ^4^ Purine derivatives—acyclovir (antiviral) and other purine-containing compounds. ^5^ Pyrimidine derivatives—methyluracil (2,4-dioxopyrimidine) and its analogues with wound-healing activity. ^6^ Other heterocycles—morpholine (in certain antibiotics), thiazolidinones, furans (nitrofural), indoles (indomethacin), quinolines (ciprofloxacin in ophthalmic ointments), benzothiazines and others.

**Table 9:** Comparison of clinical approaches to topical dermatitis therapy in CIS countries.

Country	Document	Basic Therapy	Anti-Inflammatory Topical Therapy	Antimicrobial/Antiseptic Therapy	Combination Semi-solid Dosage Forms	Specific Features
Kazakhstan	Clinical protocol for atopic dermatitis, 2019	Emollients, skin care, trigger elimination	Topical glucocorticosteroids (TCS), topical calcineurin inhibitors (TCI)	Antibacterial, antiviral, antifungal agents when necessary	Mentioned in cases of complications	Emphasizes comprehensive treatment, skin-barrier restoration, and prevention of secondary infection.
Russia	Clinical guidelines for atopic dermatitis, 2024–2026	Emollients for any severity	TCS, pimecrolimus, tacrolimus, zinc pyrithione	Only when infection is confirmed	Limited use in children; only for infection	The most modern model: emphasis on evidence-based practice, TCI, biological therapy and JAK inhibitors
Belarus	Clinical protocol for dermatitis/eczema, 2022	Emollients, soft paraffin, fats, dexpanthenol	TCS according to severity, pimecrolimus	Combinations of corticosteroids + antibiotics/antimycotics for infection	Specific combinations are widely listed	Practice-oriented protocol with a large number of topical regimens
Ukraine	Strategy for the treatment and prevention of atopic dermatitis	Skin care, prevention of exacerbations	Topical steroids, anti-inflammatory agents	The role of fungal and bacterial infections is considered	Classical and new topical products are considered	*Emphasises* rational topical therapy and prevention
Uzbekistan	National clinical protocol for atopic dermatitis, 2025	Skin care, patient education, prevention	Topical therapy, rational skin care	Systemic antifungal therapy may be used if topical treatment is ineffective	Potentially applicable in complicated forms	A new protocol, close to the modern stepwise treatment model

**Table 10:** Comparison of Clinical Recommendations and Market Representation.

Therapeutic Direction	Role in CIS Clinical Protocols	Representation in the Kazakhstani Semi-Solid Dosage Forms Market	Representation Level	Observation
Emollients/barrier-restoring agents	Basis of therapy in all protocols	Limited reflection in the drug registry; partly classified as cosmetic products	Limited	Clinical recommendations place greater emphasis on emollients than is reflected in the registered medicinal products.
Topical corticosteroids (TCS)	Main anti-inflammatory component	High representation	High	High representation in both clinical recommendations and the registry.
Topical calcineurin inhibitors (TCI)	Important steroid-sparing option	Limited representation	Limited	Limited representation in the registry relative to their role in the reviewed clinical recommendations.
Antibacterial/antiseptic agents	Prescribed for secondary infection	13.5%	Moderate	Moderately represented in the registry.
Combination products	Recommended only in cases of complications/infection	6.7%	Limited	Complex topical products show limited representation
Wound-healing/reparative agents	Important for barrier restoration and damaged skin repair	5.0%	Very limited	The largest difference between their prominence in the reviewed clinical recommendations and their representation in the registry.
DHPM-thione	Not represented in clinical recommendations or the registry; currently considered a subject for future pharmaceutical research.	0%	Not identified	Potential subject for future research

**Table 11:** Structural characteristics of the semi-solid dosage forms market in the Republic of Kazakhstan.

Parameter	Value	Interpretation
Total medicinal products in the RK register	6530	Overall base of registered products
Semi-solid dosage forms	1046	16.0% of all registered medicinal products
Ointments	413/39.5%	Dominant dosage form
Gels	302/28.9%	Second most represented form
Creams	266/25.4%	Third most represented form
Ointments + gels + creams	981/93.8%	Very high market concentration
Liniments	26/2.5%	Low representation
Plasters	22/2.1%	Limited development of alternative delivery systems
Pastes	17/1.6%	Minimal share
MCR_3_	93.8%	Very high concentration
Shannon Index	1.26	Moderate structural diversity
TMI	56.4%	High but incomplete technological maturity
FIR	1.29	Moderate predominance of modern forms

**Table 12:** Therapeutic structure of the semi-solid dosage forms market in the Republic of Kazakhstan.

Therapeutic Category	Market Share, %	Relative Position	Interpretation
Anti-inflammatory drugs	28.0	1st place	Most saturated segment
Antibacterial drugs	13.5	2nd place	Moderately saturated segment
Combination products	6.7	3rd place	Limited development of combination therapy
Wound-healing drugs	5.0	4th place	Pronounced deficit
DHPM-thione	0	absent	Not identified in the registry

**Table 13:** Therapeutic gap ratios.

Comparison	TGR	Interpretation
Anti-inflammatory/Wound-healing	5.6	Anti-inflammatory drugs represented 5.6× more frequently
Antibacterial/Wound-healing	2.7	Antibacterial drugs represented 2.7× more frequently
Anti-inflammatory/Combination	4.18	Anti-inflammatory drugs represented 4.18× more frequently
Antibacterial/Combination	2.01	Antibacterial drugs represented 2.01× more frequently
Combination/Wound-healing	1.34	Combination products represented 1.34× more frequently

**Table 14:** Comparison of pathogenetic need and actual market structure.

Key Therapeutic Target in Wound Care	Conditional Clinical Significance	Actual Market Share in Kazakhstan	Observation
Inflammation control	High	28.0%	Most frequently represented
Infection control	High	13.5%	Moderately represented
Regeneration stimulation	High	5.0%	Less frequently represented
Multitargeted therapy	Very high	6.7%	Limited representation
Novel heterocyclic APIs for combined action	High	0% for DHPM-thione	Not identified

**Table 15:** Regional comparison of semi-solid dosage forms and wound-healing products.

Parameter	Kazakhstan	Ukraine	Belarus	Interpretation	Source(s)
Share of soft forms among all drugs	16.0%	Data for the entire market not specified	About 5%	In Kazakhstan, the segment is quantitatively significant	KZ: [39,40,41]; UA: [49] BY: [50]
Share of wound-healing products within soft forms	5.0%	*Specialised* wound-care market: soft forms 50.4%	Data limited	In Kazakhstan, the wound-healing segment is less frequently represented	KZ: [39,40,41]; UA: [49]
Share of liquid wound-care forms	Not *analysed*	48.0%	Data limited	In Ukraine, the wound-healing market is more diversified	UA: [49]
Share of solid wound-care forms	Not *analysed*	1.6%	Data limited	Solid forms are minimal	UA: [49]
Share of domestic wound-care products	Limited	About 82%	Limited	Kazakhstan shows lower localisation	KZ: [39,40,41,51]; UA: [49]
Complex delivery systems	Plasters 2.1%, hydrogels limited	Developing	Plasters and TTS imported	Technological niche *characterises* Kazakhstan	KZ: [39,40,41]; BY: [50]

**Table 16:** Kazakhstan and Russia as regional models of the pharmaceutical market.

Parameter	Kazakhstan	Russia	Conclusion
Overall market scale	Substantially smaller	2.2 trillion roubles (2023)	Russia is a more mature and larger market
Market growth	Growing but remains import-dependent	+60.1% (2019–2023)	Both markets are growing, but Russia’s scale is larger
Share of domestic drugs	About 13.1% (literature data)	36.8% in monetary terms; 62.9% in units	Kazakhstan is more import-dependent
Share of imported drugs	About 86.9% (literature data)	Lower than in Kazakhstan	Import substitution in Russia is more advanced
Local manufacturers of soft forms	Limited	Numerous	Russia has a stronger manufacturing base
Combination topical products	6.7% in Kazakhstan	Reported to be more widely represented	Kazakhstan shows limited representation
DHPM/DHPM-thione products	0%	No commercial segment data available	The class is not identified in Kazakhstan

**Table 17:** International WHO context and its significance for the Kazakhstani semi-solid dosage forms market.

WHO/International Context Indicator	Value	Significance for This Study
Annual deaths from AMR	>1 million	Highlights the importance of continued antimicrobial research
Projected deaths from AMR by 2050	39 million	High importance of infection prevention and control
Antibacterial agents in WHO 2025 clinical pipeline	90	Global search for new antibacterial solutions
Traditional agents	50	Dependence on classical antibiotics persists
Non-traditional agents	40	Growing interest in novel mechanisms and platforms
Share of innovative traditional BPP agents without known cross-resistance	5 out of 27, or 19%	Innovative pipeline remains limited
Pipeline insufficiency	*Emphasised* by WHO	Provides context for continued research into new antibacterial approaches

**Table 18:** Relationship between WHO AMR recommendations and identified market gaps.

WHO Strategy Component	Significance for Wound Care	Market Status in Kazakhstan	Conclusion
Rational antibiotic use	Reduction in unnecessary antibiotic therapy	Antibacterial drugs—13.5%	An antibacterial segment is represented in the registry.
Bacterial infection diagnostics	Therapy selection based on data	Not evaluated in the register	“Diagnostics + rational topical therapy” The registry analysis did not evaluate diagnostic practices.
Novel mechanisms of action	Overcoming the limitations of classical antibiotics	DHPM-thione = 0%	Innovative class not identified
Multitargeted approaches	Inflammation + infection + regeneration	Combination products—6.7%	Limited coverage
Accessibility in LMIC/resource-limited countries	Local R&D and manufacturing	High import dependence of Kazakhstan	Development of domestic products may be relevant

**Table 19:** Representation of heterocyclic classes in the semi-solid dosage forms market.

Chemical Class	Number of Drugs	Share of Soft Forms, %
All heterocycles	490	55.0
Imidazoles	112	12.6
Triazoles	58	6.5
Purines	42	4.7
Pyridines	35	3.9
Pyrimidines	18	2.0
DHPM	0	0
DHPM-thione	0	0

**Table 20:** **Comparative positioning of DHPM-thione relative to other segments.**

Segment	Market Share, %	Therapeutic Representation	Innovation Potential	Strategic Relevance	Position
NSAIDs	28.0	High	Limited	Moderate	Mature, saturated segment
Antibacterials	13.5	Moderate	Limited	High	Important but traditional segment
Combination products	6.7	Limited	Moderate	High	Potential growth segment
Wound-healing	5.0	Limited	High	High	Less developed niche
DHPM-thione	0	Not identified	High	Very high	Unoccupied research area

**Table 21:** Inclusion and exclusion criteria for drugs in the study.

Criterion	Category	Number of Entries
Included	Ointments	413
Included	Gels	302
Included	Creams	266
Included	Liniments	26
Included	Plasters	22
Included	Pastes	17
Total included	Semi-solid dosage forms	1046
Excluded	Tablets, capsules, solutions for systemic use	Excluded before analysis
Excluded	Injectable drugs	Excluded before analysis
Excluded	Medical devices	Fully excluded
Excluded	Veterinary drugs	Fully excluded
Excluded	Technical entries and duplicates	Removed at data cleaning stage

**Note:** The excluded categories were present in the original State Register but were excluded during the screening stage because they did not meet the predefined inclusion criteria for topical semi-solid dosage forms. It should be noted that the unit of analysis was the registration record for a medicinal product, not the unique international nonproprietary name. In cases where the same active ingredient was registered by different manufacturers or in different dosage forms, each registration record was counted separately as an independent market unit Accordingly, the final analytical dataset comprised 1046 registration records of topical semi-solid dosage forms.

**Table 22:** Pharmacotherapeutic classification scheme applied in the study.

Pharmacotherapeutic Group	Main ATC Codes	Examples of Active Substances	Number of Products (n)
Wound-healing and reparative agents	D03A, D03AX	Dexpanthenol, methyluracil, sea buckthorn oil, deproteinised haemoderivative of calf blood, solcoseryl, actovegin	**68**
Antibacterial drugs for topical use	D06A, D06AX	Mupirocin, chloramphenicol, tetracycline, fusidic acid, gentamicin, erythromycin, clindamycin, ofloxacin, tyrothricin, sulfanilamide, nitrofural	**209**
Antifungal drugs	D01A, D01AE	Clotrimazole, ketoconazole, terbinafine, miconazole, econazole, sertaconazole, bifonazole, natamycin, isoconazole, fenticonazole, oxiconazole, tioconazole, naftifine, ciclopirox, flutrimazole, butoconazole	**177**
Antiseptic agents	D08A	Chlorhexidine, povidone-iodine, silver-containing drugs, benzalkonium chloride, brilliant green	**42**
Anti-inflammatory drugs (NSAIDs)	M02AA, M02AX	Diclofenac, ketoprofen, ibuprofen, piroxicam, indomethacin, nimesulide, aceclofenac, etofenamate, ketorolac, naproxen, dexketoprofen, phenylbutazone	**302**
Corticosteroids for topical use	D07A	Methylprednisolone aceponate, betamethasone, fluocinolone acetonide, hydrocortisone, mometasone, triamcinolone, alclometasone, clobetasol, desonide, prednisolone	**139**
Combination products	D07C, D07X, D06C and others	Combinations of antibiotics, corticosteroids, antiseptics and reparative components	**71**
Other categories	—	Antiviral agents (acyclovir, penciclovir, ganciclovir), hormonal products (estradiol, progesterone, testosterone), homeopathic and unclassified agents	**38**
**Total**			**1046**

**Table 23:** Criteria used for the structural-chemical classification of active pharmaceutical ingredients.

Category	Structural Criterion	Examples of Substances
Heterocyclic compounds	Presence of any heterocyclic ring	Metronidazole, clotrimazole, acyclovir
Pyridine derivatives	Presence of a pyridine ring	Niacin, pyridoxine
Pyrimidine derivatives	Presence of a pyrimidine ring	Methyluracil
Imidazole derivatives	Presence of an imidazole ring	Clotrimazole, metronidazole
Triazole derivatives	Presence of a triazole ring	Fluconazole
Purine derivatives	Presence of a purine core	Acyclovir
Quinoline derivatives	Presence of a quinoline core	Dequalinium chloride and others
Dihydropyrimidine derivatives	Presence of a 3,4-dihydropyrimidine ring	Identified separately
Dihydropyrimidine-2(1H)-thione derivatives	Presence of a DHPM-thione ring	Identified separately

**Table 24:** Summary of descriptive indicators used in the analysis.

Indicator	Purpose	Rationale
MCR_3_	Measure of market concentration	Standard, widely used indicator; provides simple quantification of dominance by top categories
Shannon Index (H)	Measure of diversity across dosage forms	Standard diversity measure; adapted from ecology; quantifies uniformity of distribution
TMI (Technological Maturity Index)	Proportion of modern dosage forms	Descriptive structural indicator; reflects the proportion of dosage forms operationally classified as modern within the descriptive framework of the study. Should not be interpreted as a validated measure of innovation or clinical superiority.
FIR (Form Innovation Ratio)	Relative proportion of modern vs. traditional forms	Complements TMI; provides ratio-based comparison
TGR (Therapeutic Gap Ratio)	Difference in representation between therapeutic categories	Simple ratio-based measure; transparent and interpretable

## Data Availability

The data presented in this study are included in the article. Further inquiries can be directed to the corresponding authors.

## Abbreviations

The following abbreviations are used in this manuscript:

AMR	Antimicrobial Resistance
API	Active Pharmaceutical Ingredient
ATC	Anatomical Therapeutic Chemical Classification System
CAGR	Compound Annual Growth Rate
CIS	Commonwealth of Independent States
COX-2	Cyclooxygenase-2
DFU	Diabetic Foot Ulcer
DHPM	Dihydropyrimidine
EAEU	Eurasian Economic Union
FIR	Form Innovation Ratio
GMP	Good Manufacturing Practice
IL-6	Interleukin-6
INN	International Nonproprietary Name
JAK	Janus Kinase
LOX	Lipoxygenase
MCR_3_	Market Concentration Ratio (top three categories)
mPGES-1	Microsomal Prostaglandin E Synthase-1
NSAIDs	Non-Steroidal Anti-Inflammatory Drugs
ROS	Reactive Oxygen Species
SPS	Strategic Priority Score
TGR	Therapeutic Gap Ratio
TMI	Technological Maturity Index (proportion of modern dosage forms; descriptive structural indicator)
TNF-α	Tumour Necrosis Factor Alpha
TS	Traditional Dosage Forms
WHO	World Health Organization

## References

[1] Machado P., Ribeiro F.N., Giublin F.C.W., Mieres N.G., Tonin F.S., Pontarolo R., Sari M.H.M., Lazo R.E.L., Ferreira L.M. (2025). Next-Generation Wound Care: A Scoping Review on Probiotic, Prebiotic, Synbiotic, and Postbiotic Cutaneous Formulations. Pharmaceuticals.

[2] Saeed S., Martins-Green M. (2024). Assessing Animal Models to Study Impaired and Chronic Wounds. Int. J. Mol. Sci..

[3] Sen C. (2023). Human Wound and Its Burden: Updated 2022 Compendium of Estimates. Adv. Wound Care.

[4] Schilrreff P., Alexiev U. (2022). Chronic Inflammation in Non-Healing Skin Wounds and Promising Natural Bioactive Compounds Treatment. Int. J. Mol. Sci..

[5] Raziyeva K., Kim Y., Zharkinbekov Z., Kassymbek K., Jimi S., Saparov A. (2021). Immunology of Acute and Chronic Wound Healing. Biomolecules.

[6] McDermott K., Fang M., Boulton A., Selvin E., Hicks C. (2022). Etiology, Epidemiology, and Disparities in the Burden of Diabetic Foot Ulcers. Diabetes Care.

[7] Armstrong D., Tan T., Boulton A., Bus S. (2023). Diabetic Foot Ulcers. JAMA.

[8] Wernecke J., Wernecke M., Ebenau O., Spruth B., Krämer M., Vogelmann T., Zöllner Y. (2025). Epidemiology and the Medical Burden of Diabetic Foot Ulcers Especially in Patients With Infection—A Population-Based Analysis From Germany. Int. Wound J..

[9] Markiewicz-Gospodarek A., Kozioł M., Tobiasz M., Baj J., Radzikowska-Büchner E., Przekora A. (2022). Burn Wound Healing: Clinical Complications, Medical Care, Treatment, and Dressing Types: The Current State of Knowledge for Clinical Practice. Int. J. Environ. Res. Public Health.

[10] Yakupu A., Zhang J., Dong W., Song F., Dong J., Lu S. (2022). The epidemiological characteristic and trends of burns globally. BMC Public Health.

[11] Gillespie B.M., Harbeck E., Rattray M., Liang R., Walker R., Latimer S., Thalib L., Andersson A.E., Griffin B., Ware R. (2021). Worldwide incidence of surgical site infections in general surgical patients: A systematic review and meta-analysis. Int. J. Surg..

[12] Richard M., Paul C., Nijsten T., Gisondi P., Salavastru C., Taieb C., Trakatelli M., Puig L., Stratigos A. (2022). EADV burden of skin diseases project team. Prevalence of most common skin diseases in Europe: A population-based study. J. Eur. Acad. Dermatol. Venereol..

[13] Ujiie H., Rosmarin D., Schön M., Ständer S., Boch K., Metz M., Maurer M., Thaci D., Schmidt E., Cole C. (2022). Unmet Medical Needs in Chronic, Non-communicable Inflammatory Skin Diseases. Front. Med..

[14] Xu J., Chang L., Xiong Y., Peng Q. (2024). Chitosan-Based Hydrogels as Antibacterial/Antioxidant/Anti-Inflammation Multifunctional Dressings for Chronic Wound Healing. Adv. Healthc. Mater..

[15] Cavallo I., Sivori F., Mastrofrancesco A., Abril E., Pontone M., Di Domenico E.G., Pimpinelli F. (2024). Bacterial Biofilm in Chronic Wounds and Possible Therapeutic Approaches. Biology.

[16] Martins-Green M., Kim J., Aziz K. (2025). The Impact of the Skin Microbiome and Oxidative Stress on the Initiation and Development of Cutaneous Chronic Wounds. Antioxidants.

[17] Wassif R.K., Shamma R., El-Hoffy N.M., El-Kayal M. (2025). Recent Advances in the Local Drug Delivery Systems for Diabetic Wound Healing: A Comprehensive Review. AAPS PharmSciTech.

[18] Tiwari R., Pathak K. (2023). Local Drug Delivery Strategies towards Wound Healing. Pharmaceutics.

[19] Alquraisy A., Ramadhani K., Mohammed A., Wilar G., Osman W., Elamin K., Wathoni N. (2026). Nanostructured Lipid Carrier-Gels for Wound Healing: A Narrative Review of Formulation Strategies, Mechanisms, and Translational Potential. Nanotechnol. Sci. Appl..

[20] Kazantsev V., Losseva I., Khrustalev D., Savelyev A., Yedrissov A., Khrustaleva A. (2026). Pinus sylvestris L. in Urban Forests of a Pollution Hotspot in Kazakhstan: Needle Phytochemistry, Bioactive Potential, and Implications for Phytoremediation. Forests.

[21] Costinaş S., Costinaş B. (2021). Structural peculiarities of normal skin and surrounding skin from removed melanocytic nevi. Med. Ecol..

[22] Xu W., Xu T., Yu L., Ning X., Zhang C., Yi B., Dai W., Zhu Z., Zhao H. (2025). Nanofibrous dressings incorporating a synergistic antibacterial-anti-inflammatory effect for infected wound healing. Mater. Today Bio.

[23] Hu S., Zhong W., Pei Y., Zhou Y., Wang J., Deng X., Teng Z., Xu L. (2025). Synergistic antibacterial and anti-inflammatory potentials of dual-loaded self-healing hydrogel for methicillin-resistant Staphylococcus aureus-infected wound healing. J. Pharm. Anal..

[24] Ren M., Wang X., Ouyang X., Ling J., Wang N. (2024). Protocatechuic acid grafted chitosan/oxidized glucomannan hydrogel with antimicrobial and anti-inflammatory effects for enhancing wound repair. Int. J. Biol. Macromol..

[25] Domínguez-Robles J., Cuartas-Gómez E., Dynes S., Utomo E., Anjani Q.K., Detamornrat U., Donnelly R.F., Moreno-Castellanos N., Larrañeta E. (2023). Poly(caprolactone)/lignin-based 3D-printed dressings loaded with a novel combination of bioactive agents for wound-healing applications. Sustain. Mater. Technol..

[26] Comino-Sanz I.M., Lopez-Franco M., Castro B., Pancorbo-Hidalgo P. (2021). The Role of Antioxidants on Wound Healing: A Review of the Current Evidence. J. Clin. Med..

[27] Savelyev A., Khrustalev D., Losseva I., Yedrissov A., Khrustaleva A., Sofiya S., Kiikbayev M., Polina R., Vladimir K. (2026). Structural and Functional Aspects of DHPM-Thiones and Their Derivatives: A Critical Review of Pharmaceutical Potential. Pharmaceuticals.

[28] Rashid H., Martines M., Duarte A., Jorge J., Rasool S., Muhammad R., Ahmad N., Umar M.N. (2021). Research developments in the syntheses, anti-inflammatory activities and structure–activity relationships of pyrimidines. RSC Adv..

[29] Nadar S., Khan T. (2021). Pyrimidine: An elite heterocyclic leitmotif in drug discovery-synthesis and biological activity. Chem. Biol. Drug Des..

[30] Al-Wahaibi L., Elshamsy A.M., Ali T., Youssif B.G.M., Bräse S., Abdel-Aziz M., El-Koussi N.A. (2024). Design and synthesis of new dihydropyrimidine/sulphonamide hybrids as promising anti-inflammatory agents via dual mPGES-1/5-LOX inhibition. Front. Chem..

[31] Tylińska B., Janicka-Kłos A., Gębarowski T., Nowotarska P., Plińska S., Wiatrak B. (2024). Pyrimidine Derivatives as Selective COX-2 Inhibitors with Anti-Inflammatory and Antioxidant Properties. Int. J. Mol. Sci..

[32] Abbas M., Arshad N. (2024). Synthesis, highly potent α-glucosidase inhibition, antioxidant and molecular docking of various novel dihydropyrimidine derivatives to treat diabetes mellitus. Bioorganic Med. Chem. Lett..

[33] Cai X., Cai J., Fang L., Xu S., Zhu H., Wu S., Chen Y., Fang S. (2024). Design, synthesis and molecular modeling of novel D-ring substituted steroidal 4,5-dihydropyrazole thiazolinone derivatives as anti-inflammatory agents by inhibition of COX-2/iNOS production and down-regulation of NF-κB/MAPKs in LPS-induced RAW264.7 macrophage cells. Eur. J. Med. Chem..

[34] Nawal N., Chawla P.A., Virendra S., Chawla V. (2024). 3,4-Dihydropyrimidine-2(1H)-one/thione Derivatives as Anti-inflammatory and Antioxidant Agents: Synthesis, Biological Activity, and Docking Studies. Curr. Org. Chem..

[35] Khrustalev D.P., Khamzina G.T., Fazylov S.D., Muldakhmetov Z.M. (2008). Microwave activation in the synthesis of nitrogen heterocycles. N-oxides of pyridine series. Russ. J. Gen. Chem..

[36] Khrustalev D., Yedrissov A., Khrustaleva A., Mustafin M., Bekisheva K. (2022). Synthesis of anti-tuberculosis drugs in a microwave flow reactor. Mater. Today Proc..

[37] Zhang Y., Shen Z., Pan B., Lu X., Chen M. (1995). Research on the Synthesis of 1,4-Dihydropyridines Under Microwave. Synth. Commun..

[38] Khrustalev D., Yedrissov A., Vetrova A., Khrustaleva A. (2020). Synthesis of 2,7-dibromo-9H-carbazole and its N-alkylation under microwave activation conditions in a flow-type microwave reactor. Mater. Today Proc..

[39] Khrustaleva A., Yedrissov A., Khrustalev D., Losseva I., Lavrinenko A., Savelyev A., Kazantsev V., Kiikbayev M., Rusyaeva P., Perepelitsyna K. (2026). Antibiotic-Loaded PLA Composites for Local Prevention of Implant-Associated Infections: Comparative Evaluation Against Reference Strains and Clinical Isolates. Antibiotics.

[40] Talgaeva S.B., Nurpeisova N.K., Saburova L.N., Utegenova A.T. (2024). Marketing Research of the Wound-Healing Gels Market in the Republic of Kazakhstan. Pharm. Kazakhstan.

[41] Akhmetova A., Saliev T., Umbayev B., Baibekov I., Ilderbayev O. (2015). Current State of the Treatment of Chronic Wounds in Kazakhstan: A Literature Review. Russ. Open Med. J..

[42] World Health Organization (2025). Global Antimicrobial Resistance Surveillance System (GLASS) Report: Early Implementation 2025.

[43] Probst A., Günther B., Woodmansey E., Bruwer F., Smith G., Woo K., Hoxha K., Idensohn P., Ramos P., Lakshmanan V. (2025). Healthcare Practitioners’ Perspectives on Infection Management, Antimicrobial Resistance and Stewardship in Wound Care Practice. J. Wound Care.

[44] Glushchenko O.M., Polova Z.H.M. (2019). Market analysis of wound healing semisolid drugs. Pharm. Rev..

[45] National Register of Medicinal Products of the Republic of Kazakhstan. National Center for Expertise of Medicines and Medical Devices, Ministry of Health of the Republic of Kazakhstan. https://register.ndda.kz/#/reestr.

[46] Bunn D.W., Salo A.A. (1993). Forecasting with Scenarios. Eur. J. Oper. Res..

[47] Stewart A.J.A., Stow M.T. (2023). Modeling a Scenario-Based Approach for Foresight Pharmaceuticals. Eur. J. Inf. Technol. Comput. Sci..

[48] Tonin F., Aznar-Lou I., Pontinha V., Pontarolo R., Fernandez-Llimos F. (2021). Principles of Pharmacoeconomic Analysis: The Case of Pharmacist-Led Interventions. Pharm. Pract..

[49] Patanwala A.E., Narayan S.W., Haas C.E., Abraham I., Sanders A.B., Erstad B.L. (2021). Proposed Guidance on Cost-Avoidance Studies in Pharmacy Practice. Am. J. Health-Syst. Pharm..

[50] Su H.-Y., Yang C.-Y., Ou H.-T. (2023). Cost-Effectiveness of Novel Macrophage-Regulating Treatment for Wound Healing in Patients with Diabetic Foot Ulcers from the Taiwan Health Care Sector Perspective. JAMA Netw. Open.

[51] Duko A.A., Hutkina G.A., Rzheuski S.E., Atroschenko V.A. (2022). Analysis of the nomenclature of semi-solid dosage forms registered in the Republic of Belarus. Vestn. Vitebsk State Med. Univ..

[52] IndexBox (2026). Kazakhstan Topical Drugs (Dermatologicals) Market: CDMO Industry Analysis and Forecast 2026–2035.

[53] Alfarm (2024). Statistical Report on the Pharmaceutical Market of the Russian Federation 2023.

[54] Sultanov R. (2024). Farmatsevticheskii Sektor Kazakhstana: Stremitel’noe Razvitie Eksportnogo Potentsiala i Novye Vyzovy [Pharmaceutical Sector of Kazakhstan: Rapid Development of Export Potential and New Challenges]. EC[ON]OMY. https://economykz.org/farmaczevticheskij-sektor-kazahstana/.

[55] Ulysmedia Analytical Service (2025). Pharmaceutical Market of Kazakhstan: Results of 2024 and Development Trends. Ulysmedia.kz. https://ulysmedia.kz.

[56] Zhakipbekov K., Posylkina O., Zhumabayev N., Datkhayev U., Zhumabayev N., Almurzaeva A., Mukanova A. (2023). Analysis of the Current State of the Pharmaceutical Market of the Republic of Kazakhstan. Sci. Rise Pharm. Sci..

[57] Li J., Zhang Y., Wang H., Chen X., Liu Z., Wu Q. (2025). Bacteria-Responsive Nanoplatform with Dihydropyrimidine Fragments for Infected Diabetic Wound Healing via ROS Scavenging and Biofilm Disruption. Acta Biomater..

[58] Chandravarkar A., Aneeja T., Anilkumar G. (2023). Advances in Biginelli reaction: A comprehensive review. J. Heterocycl. Chem..

[59] Sarvaiya B.H., Vaja P.I., Paghdar N.A., Ghelani S.M. (2024). Medicinal perspective of a promising scaffold—Dihydropyrimidinones: A review. J. Heterocycl. Chem..

[60] Transparency Market Research (2025). Topical Drugs Market—Global Industry Analysis, Size, Share, Growth, Trends, and Forecast, 2025–2035.

[61] Stratistics Market Research Consulting (2025). Advanced Drug Formulation Market—Global Forecast 2025–2032.

[62] Kaliyeva D., Turgambayeva A., Kerimbayeva Z. (2022). Registration procedure of generic drugs in the Republic of Kazakhstan and Europe: Review. J. Clin. Med. Kaz..

[63] García-Arieta A., Gordon J., Gwaza L., Merino V., Mangas-Sanjuán V. (2023). Regulatory Requirements for the Development of Second-Entry Semisolid Topical Products in the European Union. Pharmaceutics.

[64] Miranda M., Volmer Z., Cornick A., Goody A., Cardoso C., Pais A.A., Brown M., Vitorino C. (2024). In vitro studies into establishing therapeutic bioequivalence of complex topical products: Weight of evidence. Int. J. Pharm..

[65] Ministry of Health of the Republic of Kazakhstan Development Plan of the Ministry of Health of the Republic of Kazakhstan for 2023–2027; Approved by Order of the Minister of Health of the Republic of Kazakhstan No. 64 Dated 27 January 2023, as Amended by Order No. 964 Dated 31 December 2024. https://www.gov.kz/memleket/entities/dsm/documents/details/415138?lang=ru.

[66] Ministry of Health of the Republic of Kazakhstan (2025). Report on the Implementation of the Development Plan of the Ministry of Health of the Republic of Kazakhstan for 2023–2027: Reporting Period—2024.

[67] Hollingworth S., Kairuz T. (2021). Measuring Medicine Use: Applying ATC/DDD Methodology to Real-World Data. Pharmacy.

[68] Nantongo H., Batwaala V., Nambasa V., Mukonzo J. (2022). Application of the Anatomical Therapeutic Chemical Classification System/Defined Daily Doses: Challenges and Way Forward for Resource-Limited Countries. J. Clin. Pharm. Ther..

[69] Ravela R., Lyles A., Airaksinen M. (2022). National and Transnational Drug Shortages: A Quantitative Descriptive Study of Public Registers in Europe and the USA. BMC Health Serv. Res..

[70] Green A., Lyus R., Ocan M., Pollock A., Brhlikova P. (2023). Registration of Essential Medicines in Kenya, Tanzania and Uganda: A Retrospective Analysis. J. R. Soc. Med..

[71] Kovaleva E.A., Nemyatykh O., Narkevich I., Makhova O.A. (2024). Analysis of Pediatric Medicines Pharmaceutical Market Segment. Med. Technol. Assess. Choice.

[72] Narkevich I., Nemyatykh O., Siukaeva D.D., Tsitlionok E.A., Lisacenco V.O., Grinyuk A.S. (2020). Multi-Vector Analysis of the Market for Drugs Used to Treat Hepatitis C. Pharm. Formulas.

[73] Mehta A., Farooqui H.H., Selvaraj S. (2016). A Critical Analysis of Concentration and Competition in the Indian Pharmaceutical Market. PLoS ONE.

[74] Kvålseth T.O. (2022). Measurement of Market (Industry) Concentration Based on Value Validity. PLoS ONE.

[75] Silalahi M., Nisyawati, Walujo E.B., Supriatna J., Mangunwardoyo W. (2015). The Local Knowledge of Medicinal Plants Trader and Diversity of Medicinal Plants in the Kabanjahe Traditional Market, North Sumatra, Indonesia. J. Ethnopharmacol..

[76] Asra R., Ihsan M., Andriani F., Silalahi M., Fijridiyanto I., Maryani A. (2023). Diversity of Medicinal Plants Sold in the Traditional Markets in Jambi, Indonesia. Biodiversitas J. Biol. Divers..

[77] Vennemann M., Ruland V., Kruse J., Harloff C., Trübel H., Gielen-Haertwig H. (2019). Future Unmet Medical Need as a Guiding Principle for Pharmaceutical R&D. Drug Discov. Today.

[78] Kusynová Z., Pauletti G., van den Ham H.A., Leufkens H.G.M., Mantel-Teeuwisse A.K. (2022). Unmet Medical Need as a Driver for Pharmaceutical Sciences—A Survey among Scientists. J. Pharm. Sci..

[79] Schramm L., Monsefi N., Hüser J., Trübel H., Mondritzki T. (2022). A Novel Approach for Quantification of the Future Unmet Medical Need in Right Ventricular Dysfunction. Drug Discov. Today.

[80] Drews J., Ryser S. (1996). Innovation Deficit in the Pharmaceutical Industry. Ther. Innov. Regul. Sci..

[81] Khanna I. (2012). Drug Discovery in Pharmaceutical Industry: Productivity Challenges and Trends. Drug Discov. Today.

[82] Pammolli F., Magazzini L., Riccaboni M. (2011). The Productivity Crisis in Pharmaceutical R&D. Nat. Rev. Drug Discov..

[83] Dorohan-Pysarenko L., Yehorova O., Yasnolob I. (2023). Methodological and Applied Aspects of Using the BCG Matrix. Mark. Infrastruct..

[84] Chiu C.-C., Lin K.-P. (2019). Rule-Based BCG Matrix for Product Portfolio Analysis. Software Engineering, Artificial Intelligence, Networking and Parallel/Distributed Computing.

[85] MacMillan I.C., Hambrick D.C., Day D.L. (1982). The Product Portfolio and Profitability—A PIMS-Based Analysis of Industrial-Product Businesses. Acad. Manag. J..

